# Potentiation of combined p19Arf and interferon-beta cancer gene therapy through its association with doxorubicin chemotherapy

**DOI:** 10.1038/s41598-022-17775-y

**Published:** 2022-08-10

**Authors:** Ruan F. V. Medrano, Thiago A. Salles, Rafael Dariolli, Fernanda Antunes, Valker A. Feitosa, Aline Hunger, João P. P. Catani, Samir A. Mendonça, Rodrigo E. Tamura, Marlous G. Lana, Elaine G. Rodrigues, Bryan E. Strauss

**Affiliations:** 1 Laboratório de Vetores Virais, Centro de Investigação Translacional Em Oncologia/LIM 24, Instituto do Câncer do Estado de São Paulo (ICESP), Faculdade de Medicina, Universidade de São Paulo (FM-USP), Av. Dr. Arnaldo, 251, 8° Andar, São Paulo, SP CEP: 01246-000 Brazil; 2 Laboratório de Genética e Cardiologia Molecular/LIM 13, Instituto do Coração, FM-USP, São Paulo, SP Brazil; 3 Department of Pharmacological Sciences, Icahn School of Medicine at Mount Sinai, New York, NY 10029 USA; 4 Núcleo de Bionanomanufatura, Instituto de Pesquisas Tecnológicas (Bionano-IPT), São Paulo, SP Brazil; 5 Department of Radiation Oncology, Washington University School of Medicine, St. Louis, MO USA; 6 Department of Microbiology, Immunology and Parasitology, Paulista School of Medicine, Federal University of São Paulo (EPM-UNIFESP), São Paulo, Brazil; 7 Present Address: Department of Pathology and Immunology, Washington University School of Medicine, St. Louis, MO 63110 USA; 8 Present Address: Faculdade de Ciências Farmaceuticas, Universidade Estadual Paulista Júlio de Mesquita Filho, Araraquara, SP Brazil; 9 Present Address: Cristalia, Biotecnologia Unidade 1, Rodoviária SP 147, Itapira, SP Brazil; 10 Present Address: Vlaams Instituut Voor Biotenchnologie-UGent Center for Medical Biotechnology, Gent, Belgium; 11 Present Address: Department of Biological Sciences, Federal University of São Paulo, Diadema, SP Brazil

**Keywords:** Cell death, Cancer immunotherapy, Chemotherapy

## Abstract

Balancing safety and efficacy is a major consideration for cancer treatments, especially when combining cancer immunotherapy with other treatment modalities such as chemotherapy. Approaches that induce immunogenic cell death (ICD) are expected to eliminate cancer cells by direct cell killing as well as activation of an antitumor immune response. We have developed a gene therapy approach based on p19Arf and interferon-β gene transfer that, similar to conventional inducers of ICD, results in the release of DAMPS and immune activation. Here, aiming to potentiate this response, we explore whether association between our approach and treatment with doxorubicin (Dox), a known inducer of ICD, could further potentiate treatment efficacy without inducing cardiotoxicity, a critical side effect of Dox. Using central composite rotational design analysis, we show that cooperation between gene transfer and chemotherapy killed MCA205 and B16F10 cells and permitted the application of reduced viral and drug doses. The treatments also cooperated to induce elevated levels of ICD markers in MCA205, which correlated with improved efficacy of immunotherapy in vivo. Treatment of subcutaneous MCA205 tumors associating gene transfer and low dose (10 mg/kg) chemotherapy resulted in inhibition of tumor progression. Moreover, the reduced dose did not cause cardiotoxicity as compared to the therapeutic dose of Dox (20 mg/kg). The association of p19Arf/interferon-β gene transfer and Dox chemotherapy potentiated antitumor response and minimized cardiotoxicity.

## Introduction

Immunogenic chemotherapy relies on several distinct mechanisms to modulate the tumor microenvironment (TME) and activate the immune system^1–6^, including through the induction of immunogenic cell death (ICD), which is characterized by a series of regulated events that mediate the release of danger associated molecular patterns (DAMPS) from dying cancer cells to stimulate robust maturation of antigen presenting cells (APCs) and effective priming of CD8^+^ T cells^7^. Such events include secretion of ATP^8^, exposure of calreticulin from the endoplasmic reticulum^9^, as well as the passive release of high-mobility group box 1 (HMGB1)^10^. Even though these classical ICD markers were initially identified as a cellular response to anthracyclines, including doxorubicin (Dox)^11^, additional chemotherapeutic agents as well as other classes of ICD inducers have been identified, such as radiotherapy^10^ and hypericin-based photodynamic therapy^12^. Importantly, each of these agents differ in the induction of DAMPS both in terms of magnitude and the subset of ICD markers emitted from dying cells^13^, making the association of different ICD inducers an interesting field of investigation.

To this end, we have developed a set of replication deficient adenoviral vectors (serotype 5) that encode the p19^Arf^ (tumor suppressor protein, p53 functional partner) and murine interferon-β (IFNβ, pleiotropic immunomodulatory cytokine) cDNAs under the control of a p53 responsive promoter, named PGTxβ (or simply PG). We have shown that adenoviral vector mediated delivery of the p19^Arf^ and IFNβ (p19Arf/IFNβ) combination to tumor cells that harbor wild type p53, such as B16F10, unleashes a cell death program that displays features of necroptosis, while mediating the release of *bona fide* ICD markers as well as immune protection against a secondary tumor challenge^14–16^. Indeed, in a previous study, we explored a prophylactic vaccine model and observed that when injected during the cell death process, B16F10 mouse melanoma cells treated ex vivo with the p19Arf/IFNβ combination, elicited a CD4^+^ and CD8^+^ T cell dependent protection against fresh naive B16F10 cells injected in the opposite flank^17^. We also explored an in situ gene therapy model and obtained supporting evidence of the superior immune stimulatory capability of the p19Arf/IFNβ combination over the single use of IFNβ^18^.

Here, we propose that the efficacy of our vector-based approach could be further improved through its association with other therapies and investigated potential benefits of associating p19Arf/IFNβ gene therapy with Dox immunogenic chemotherapy, a well-known inducer of ICD^11^ frequently used for the treatment of sarcoma, lung, ovarian and other cancers^19^.

Dox is proposed to act by disrupting topoisomerase II-mediated DNA repair and by producing free radicals that inflict damage to several cellular components^19^. Additionally, as previously reported by our group and others, treatment with Dox can lead to the activation of the apoptotic p53 transcriptional pathway^20^. Even though it is an effective anticancer agent, its use is limited, in part, by the induction of severe cardiotoxicity which, depending on the dose, can evolve to chronic cardiomyopathy with a high rate of mortality^21,22^.

Our results indicate that the association of p19Arf/IFNβ with Dox (p19Arf/IFNβ + Dox) drastically enhances cell death in vitro, allowing the application of a reduced dose of adenovirus and Dox. This association also enhances the immunogenicity of treated cells by increasing the secretion of ATP and exposure of HMGB1, which in a therapeutic vaccine model resulted in superior antitumor protection when compared to the single therapy. Upon i.t treatment of established tumors, this association improved tumor control by our gene therapy, matching the survival benefit of the therapeutic, yet cardiotoxic, high dose of Dox. Moreover, pre-treatment with p19Arf/IFNβ gene therapy is able to enhance the effect of a sub-therapeutic dose of Dox, performing as well as the therapeutic dose while preserving cardiac function. Therefore, use of p19Arf/IFNβ gene therapy in association with another ICD inducer such as Dox provides important combinatorial benefits to both therapies and warrants further investigation.

## Methods

### Cell culture and cell lines

The mouse cell lines MCA205 H-2b (MCA, methylcholanthrene derived sarcoma, provided by Dr. Guido Kromer, France) and B16F10 (B16, melanoma, kindly provided by Dr. Roger Chammas, ICESP) were maintained in a humidified incubator at 37 °C with 5% CO_2_ and cultivated in Roswell Park Memorial Institute (RPMI) medium (Thermo Fisher Scientific, Waltham, MA, USA), supplemented with 10% fetal bovine serum (Invitrogen) as well as 1X Anti-Anti (Antibiotic–Antimycotic -100X, Thermo Fisher Scientific). HEK293 cells were cultivated in Dulbecco’s modified Eagle medium (both from Thermo Fisher Scientific), supplemented and maintained in the same conditions as above.

Here we use the MCA sarcoma cell line and employed an intratumoral (i.t) application model since it was demonstrated under these conditions the ability of Dox to unleash ICD and stimulate immune responses in vivo^11^. We also used the B16 cell line, as it was with this model that we revealed the cell death and immune stimulatory events of our p19Arf/IFNβ treatment. With regard to the treatment order, we based our approach on the work of Fridlender and collaborators (2010) that showed that association of an adenoviral vector encoding IFNβ with chemotherapy is more effective when gene transfer is applied first^23^.

The MCA-DEVD cell line was generated by transduction with a lentivirus reporter for caspase-3 activity and selection for puromycin resistance (0.5 μg/ml). This vector, previously described^24^, encodes a constitutively expressed luciferase-GFP protein separated from a polyubiquitin domain via a caspase-3 cleavage site and was generously provided by Dr. Chuan-Yuan Li (Department of Radiation Oncology, University of Colorado School of Medicine, Aurora, CO, USA).

### Virus construction, production and titration

Construction and production of AdRGD-PG adenoviral vectors (serotype 5) containing modification with the RGD motif in the fiber as well as the p53-responsive promoter (PGTxβ, PG) has been described previously^14^. Titration of adenoviral stocks was performed using the Adeno-X Rapid Titer Kit (Clontech, Mountain View, CA, USA) and titer yields were: AdRGD-CMV-LacZ (3.6 × 10^9^ IU/mL, infectious units/milliliter), AdRGD-PG-LUC (1 × 10^11^ IU/mL), AdRGD-PG-eGFP (5 × 10^10^ IU/mL), AdRGD-PG-p19 (1.3 × 10^10^ IU/mL) and Ad-RGD-IFNβ (5 × 10^10^ IU/mL). This biological titer was used to calculate multipilicity of infection (MOI).

### In vitro assays

MCA or B16 cells (1 × 10^5^) were plated in 6 well plates containing 1 mL of RPMI media and transduced with adenovirus at the desired MOI. After an overnight transduction period (12–16 h), 2 mL of media was added and cells kept in culture until needed. When combining adenoviral transduction with chemotherapy, Dox (doxrubicin hydrochloride, Sigma, St. Louis, MO, USA) was added immediately after the overnight transduction using the concentration indicated for each experiment. Importantly, in the Dox single treatment condition, Dox was added at the same moment as in the association group, 12 to 16 h after cell plating. After 12 h treatment with Dox (1 mg/mL) or Nutlin-3 (10 μM, Sigma), expression of eGFP from AdRGD-PG-eGFP was analyzed by flow cytometry (Attune®, Life Technologies). Cell viability was assessed by MTT assay where, 8 h after transduction in 6 well plates, 2 × 10^4^ cells/well were plated in 96 well plates, treated with Dox, and analyzed after 16 h of incubation. Non-transduced cells were used as viable control and protocol was carried out as described previously^25^. Cell cycle analysis by propidium iodide (PI) staining was carried out 72 h after p19Arf/IFNβ and Dox single treatment, as previously described^16^. Analysis of caspase 3 activity in vitro was performed 16 h after combined treatment using the CellEvent Caspase-3/7 Green Reagent (Thermo Fisher Scientific) by flow cytometry, following manufacturer’s instructions. Last, analysis of ICD markers upon p19Arf/IFNβ + Dox was conducted as detailed previously^14^. Briefly, detection of calreticulin^+^ and PI^-^ cells was made 14 h after combined treatment, by staining with a CRT-specific antibody (1:100, Novus, Biologicals, CO, USA) and after cells were washed with PBS, they were incubated with Alexa488-conjugated anti-rabbit secondary antibody (1:500, Thermo Fisher Scientific) followed by PI staining to exclude dead cells, immediately before flow cytometry. Accumulation of ATP in the cell supernatant was detected using the ENLITEN ATP Assay (Promega, Madison, WI, USA), as per the manufacturer's instructions. Luminescence was observed using a GloMax Plate Reader (Promega). HMGB1 in cell supernatant was detected by Western blot after conditioned medium was supplemented with protease inhibitor cocktail (Thermo Fisher Scientific). Then, 180 µl of the medium was concentrated (Concentrator Plus—Eppendorf, Hamburg, Germany) and subjected to western blotting. Unrelated, high molecular weight regions of the membrane were removed before detection was performed using anti-HMGB1 (Abcam ab79823, Cambridge, UK) and a secondary antibody conjugated with horseradish peroxidase before visualization using ECL (GE Healthcare, Chicago, IL, USA) and the ImageQuant LAS4000 imaging platform (GE Healthcare). See ‘Supplementary Information Westerns S2’ for original images from three independent assays. Additional Western blots were performed using cell lysates, high-molecular weight regions of the membranes were removed and then detection was performed using anti- PARP (Cell Signaling, Danvers, MA, USA, #9542), anti-Actin (Santa Cruz Biotechnology, Dallas, TX, USA, #47778), anti-Caspase 3 (Cell Signaling, #9662), anti-Tubulin (Millipore, Burlington, MA, USA, #05-829) and the appropriate secondary antibodies conjugated with horseradish peroxidase (anti-mouse—Sigma #A9044 e anti-rabbit—Sigma #A0545). See ‘Supplementary Information Westerns S2’ for original images from two independent assays.

### In vitro association of p19Arf/IFNβ and Dox

The influence of two independent variables, namely, MOI of adenoviral vectors encoding p19Arf/IFNβ and the concentration of Dox, was investigated on MCA and B16 cells using factorial experiments in five levels (Table S1), with the percentage of hypodiploid cells as the variable response. The experiments were carried out employing central composite rotational design (CCRD) where, for each cell line, a set of twelve combinatory assays containing a central composite factorial matrix plus rotation points, central points and controls was performed (Table S2, where the assays and conditions are provided in detail**)**. To better visualize the effects and interactions of MOI and Dox concentration on the percentage of hypodiploid cells, assessed by PI staining after 20 h of treatment, the results were plotted in response surface graphs.

Importantly, the statistical significance of the independent variables and their interactions was determined by Fisher’s post-test for an analysis of variance (ANOVA) and Pareto chart analysis, both at a confidence level of 95% (p ≤ 0.05). Moreover, five repetitions at the central point (CP) assays were used to minimize the error term of the ANOVA. Experimental designs, data regression and graphical analysis were performed using the *Statistica* software v.7.0 (*Statsoft,* Inc., Tulsa, OK, USA).

### Ethics statement

Both C57BL/6 and Nude mice were female, 7 weeks old, obtained from the *Centro de Bioterismo da FMUSP* and kept in the animal facility in the *Centro de Medicina Nuclear* (CMN) in SPF conditions, with food and water ad libitum. The methods are reported in accordance with ARRIVE guidelines. The well-being of the mice was constantly monitored and all methods, including vaccination protocols, in vivo gene therapy, imaging, echocardiographic assessments, anesthesia and euthanasia were carried out in accordance with relevant guidelines and regulations of Brazil and our institution whose ethics committee (Committee for the Ethical Use of Animals, CEUA, University of São Paulo School of Medicine, FMUSP) approved this project (protocol n° 165/14).

### Immunotherapy model

In the first step of the immunotherapy model, naïve C57BL/6 mice were inoculated (s.c) in the right flank (tumor challenge site) with fresh untreated MCA (2 × 10^5^) or B16 (6 × 10^4^) cells and in the second step, vaccinated (s.c) on days + 3, + 9 and + 15 with 3 × 10^5^ ex vivo treated cells applied in the left flank (vaccine site). Ex vivo treatment was carried out as follows: MCA or B16 cells were seeded in 10 cm plates with 2 mL of media and co-transduced with the AdRGD-PG-p19 and AdRGD-PG-IFNβ (MOI 500 for each) for 4 h before the addition of 8 mL of fresh media. Then, cells were kept in culture for 16 h and in the p19Arf/IFNβ + Dox or Dox groups, Dox (14 µM) was added for 6 h, until cells were harvested, washed twice with cold PBS, counted and resuspended in 100 µL of cold PBS. For the DEAD cell + GFP control group, cells were transduced with the AdRGD-PG-eGFP vector (MOI 1000) and after 16 h, harvested, washed twice with cold PBS, resuspended and lysed by three cycles of freezing and thawing.

### In vivo gene therapy and doxorubicin treatment models

MCA (2 × 10^5^) or B16 (5 × 10^5^) cells were harvested, washed twice with cold PBS, resuspended in 100 µL of PBS per mouse and then inoculated subcutaneously (s.c) in the left flank of immune competent C57BL/6 or immune deficient Balb/c Nude (*Foxn1n*) mice. While mice were not randomized after injection of cells, but there was no specific selection of animals for each treatment group. No blinding of group allocation was performed at any phase of experimentation. No animals were excluded from the data. Approximately 8 days later, palpable (60 mm^3^) tumors were treated three times, once every 2 days, with intratumoral (i.t) injections (administered with precision Hamilton glass syringes (volume 100µL) and 26G needles) of the following adenoviral vectors, AdRGD-CMV-LacZ or AdRGD-PG-LUC (4 × 10^8^ IU, resuspended in 25 µL final volume of PBS/mouse) or treated with the combination of AdRGD-PG-p19 and AdRGD-PG-IFNβ (2 × 10^8^ IU, for each vector and maintaining the 25 µL final volume per mouse). For the Dox single treatment model, chemotherapy was applied (i.t) once on day 12 with the following doses: 60, 20, 10 or 5 mg/kg (in the final volume of 30 µL of PBS/mouse). Whereas in the association model, adenoviral vectors were injected as explained above and Dox given 2 days after the last viral injection (day 14), following the injection method as the Dox single treatment group. Tumor progression was measured by calipers every two days and volume calculated as described^17^. For the survival analysis comparing C57BL/6 and Nude mice, treated mice were euthanized by anesthesia with ketamine/xylazine followed by CO_2_ inhalation when tumor volume reached 1000 mm^3^ unless otherwise noted. See figure legends for the number of animals in each experimental group.

### In vivo bioluminescence imaging

For the analysis of caspase 3 in vivo*,* MCA-DEVD tumors were treated in situ as described above and 24 and 48 h after the last treatment injection, mice were submitted to bioluminescence imagining (IVIS Spectrum, Caliper Life Science) to detect the luciferase activity from the DEVD reporter. To this end, 10 mg/kg luciferin (Promega) was administered by intraperitoneal (i.p) injection of each mouse and these were anesthetized with isoflurane (Cristalia, São Paulo, Brazil) using the Xenogen anesthesia system before imaging. Images were captured and only the strongest signal from each tumor was included in the analysis with Living Imaging 4.3 software (PerkinElmer, Waltham, MA, USA). Luciferase activity was obtained from the average radiance value [p/s/cm^2^/sr]. To calculate the fold activity overtime, average radiance values obtained for each mouse 48 h post-treatment were divided by its respective value at 24 h. Parental MCA tumors were used as negative control and no emission was detected (data not shown).

### Echocardiographic assessments

The systolic cardiac function was assessed by echocardiography. Exams were performed 10 days after treatments with AdRGD-PG-eGFP (adenovirus control), Dox 10 mg/kg, Dox 20 mg/kg and p19Arf/IFNβ + Dox 10 mg/kg. Mice were anesthetized with 1.5 to 2.5% isoflurane (in 100% oxygen ventilation). They were trichotomized and placed in supine decubitus to obtain cardiac images. Parasternal-long and short axis images were captured using VEVO 2100 ultrasound equipment (Vevo 2100 Imaging System, VisualSonics, Toronto, Canada) with a 40 MHz linear-transducer. Analyses were performed off-line using VevoCQ LV Analysis software (VisualSonics). Parameters such as systolic and diastolic volumes were calculated using Simpson’s modified algorithms present in the analysis software (parasternal-long axis images). Based on these volumes, stroke volume (μL) and left ventricle ejection fraction (LVEF, %) were calculated. Also, linear measurements were obtained from parasternal short axis images. Left ventricle shortening fraction (LVSF, %) was calculated, using systolic and diastolic diameters. Left ventricle mass (LV mass, mg) was estimated by linear measurements. Beating rate (beats per minute, BPM) was recorded directly by an animal table-ECG system connected to the VEVO 2100 system. Echocardiographic results were interpreted considering the American Society of Echocardiography recommendations concerning the mouse model^26^. All parameters were shown as the mean values of three consecutive cardiac cycles. Transthoracic echocardiography image acquisition and analysis was performed by an expert investigator who was blind to the experimental groups.

### Statistical analysis

Data are presented as mean ± SEM. Statistical differences between groups are indicated with *p* values, being *p < 0.05, **p < 0.01 and ***p < 0.001. Statistical tests are indicated in each figure legend along with the number of independent experiments performed or number (n) of mice in each group. These analyses were made using the GraphPad Prism 5 (La Jolla, CA, USA) software, with the exception of the CCRD analysis (explained above).

## Results

### Use of p19Arf/IFNβ or doxorubicin as monotherapies

Considering that the response of the MCA cell line to our AdRGD-PG vectors has not been previously studied, we first confirmed efficient transduction and p53-driven expression from the AdRGD-PG-eGFP vector upon Dox or Nutlin-3 treatment (Fig. S1a). Co-transduction with the AdRGD-PG-p19 and AdRGD-PG-IFN-β vectors (p19Arf/IFNβ) augments cell death levels in comparison with individual treatments or the GFP control vector (Fig. S1b), in agreement with our previous observations made with the B16 and LLC-1 cell lines^16,18^.

**Figure 1:**
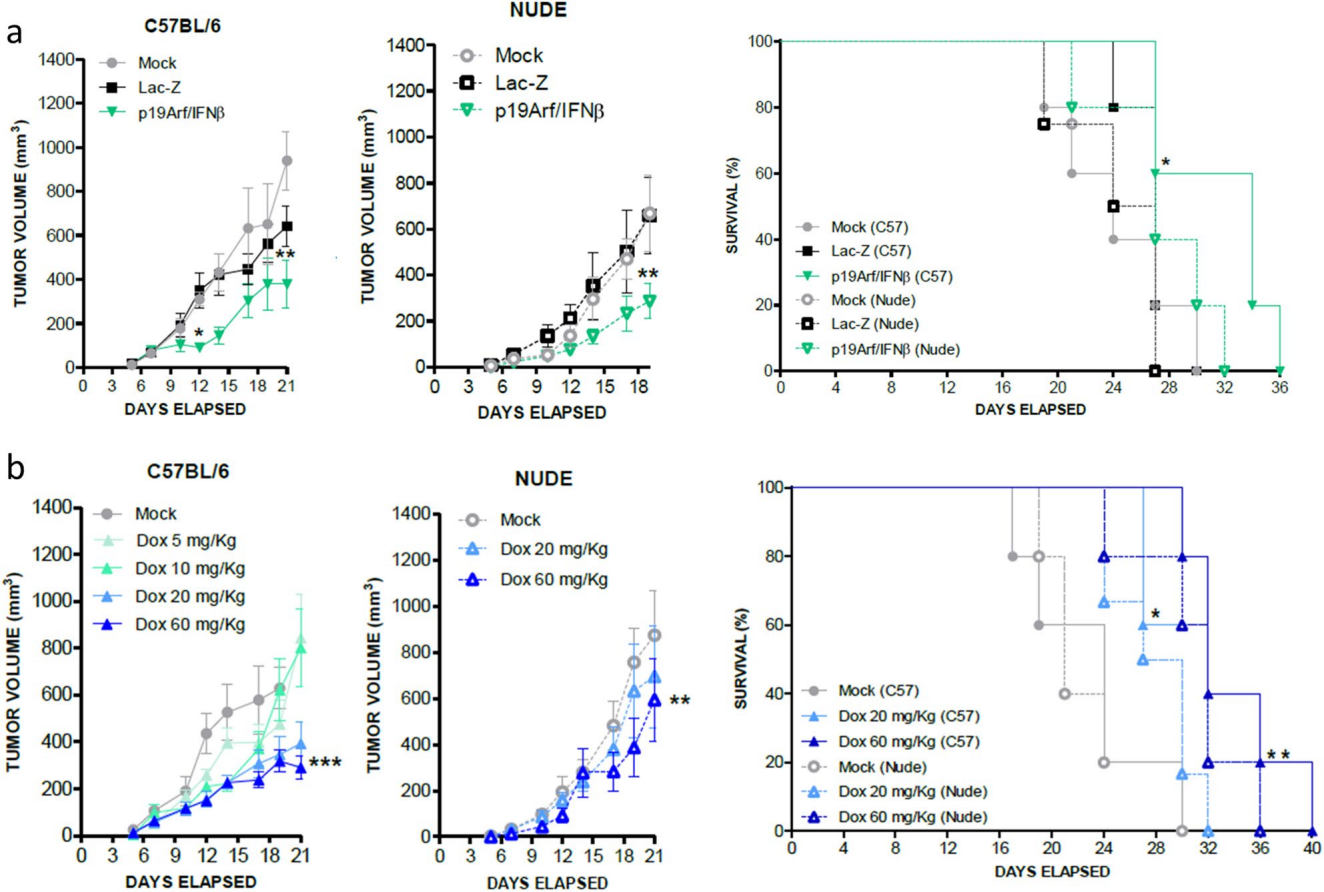
Use of p19Arf/IFNβ and doxorubicin as monotherapies inhibits progression of established MCA tumors in immune competent hosts. (**a**) Progression of MCA tumors in C57BL/6 and Nude mice upon in situ PBS (Mock), Lac-Z or p19Arf/IFNβ gene therapy. [Two-way Anova and Bonferroni post-test]. Survival analysis comparing immune competent C57BL/6 mice and immune deficient Nude mice [Log Rank Mantel-cox test, followed by Wilcoxon post-Test]. n = 5 for the p19Arf/IFNβ (C57), Lac-Z (C57), Mock (C57, p19Arf/IFNβ (Nude), n = 4 for Mock (Nude) and Lac-Z (Nude) groups. (**b**) Progression of MCA tumors treated intratumorally with PBS (Mock) or doxorubicin (Dox) and performed in C57BL/6 and Nude mice. [Two-way Anova and Bonferroni post-test]. Survival analysis comparing immune competent C57BL/6 and immune deficient Nude mice. [Log Rank Mantel-cox test followed by Wilcoxon post-Test]. n = 6 for the Mock (C57), Mock (Nude), Dox 60 mg/kg (C57) groups; n = 4 for Dox 20 mg/kg (C57), Dox 20 mg/kg (Nude) and n = 3 Dox 60 mg/kg.

Employing an in situ gene therapy model, where established MCA tumors are treated with intratumoral injections of adenoviral vectors, we next examined the antitumor efficacy of our gene therapy approach when treatment was performed in C57BL/6 immune competent mice or in T cell deficient Nude mice. Significant reduction in tumor progression was seen in either host (Fig. 1a), likely benefiting from NK cell activity as seen in our previous study^17^. Survival was improved when treatment was performed in the immune competent host, suggesting the involvement of T cells in the therapeutic response (Fig. 1a).

We also analyzed the effect of Dox as an immunogenic monotherapy. In vitro, MCA cells were readily killed by Dox in a dose responsive manner (Fig. S1c). In vivo, using a single intratumoral injection of Dox, the dose of 20 mg/kg or 60 mg/kg were shown to inhibit tumor progression and prolong survival (Fig. 1b), especially when therapy was performed in an immune competent host, as previously observed^11,27^. Since the dose of 10 mg/kg was not effective in inhibiting tumor progression, this will be considered hereafter as a sub-therapeutic dose. These results reveal the efficacy of p19Arf/IFNβ and Dox when applied as monotherapies and prompted us to investigate their association.

### In vitro association of p19Arf/IFNβ and Dox

**Figure 2:**
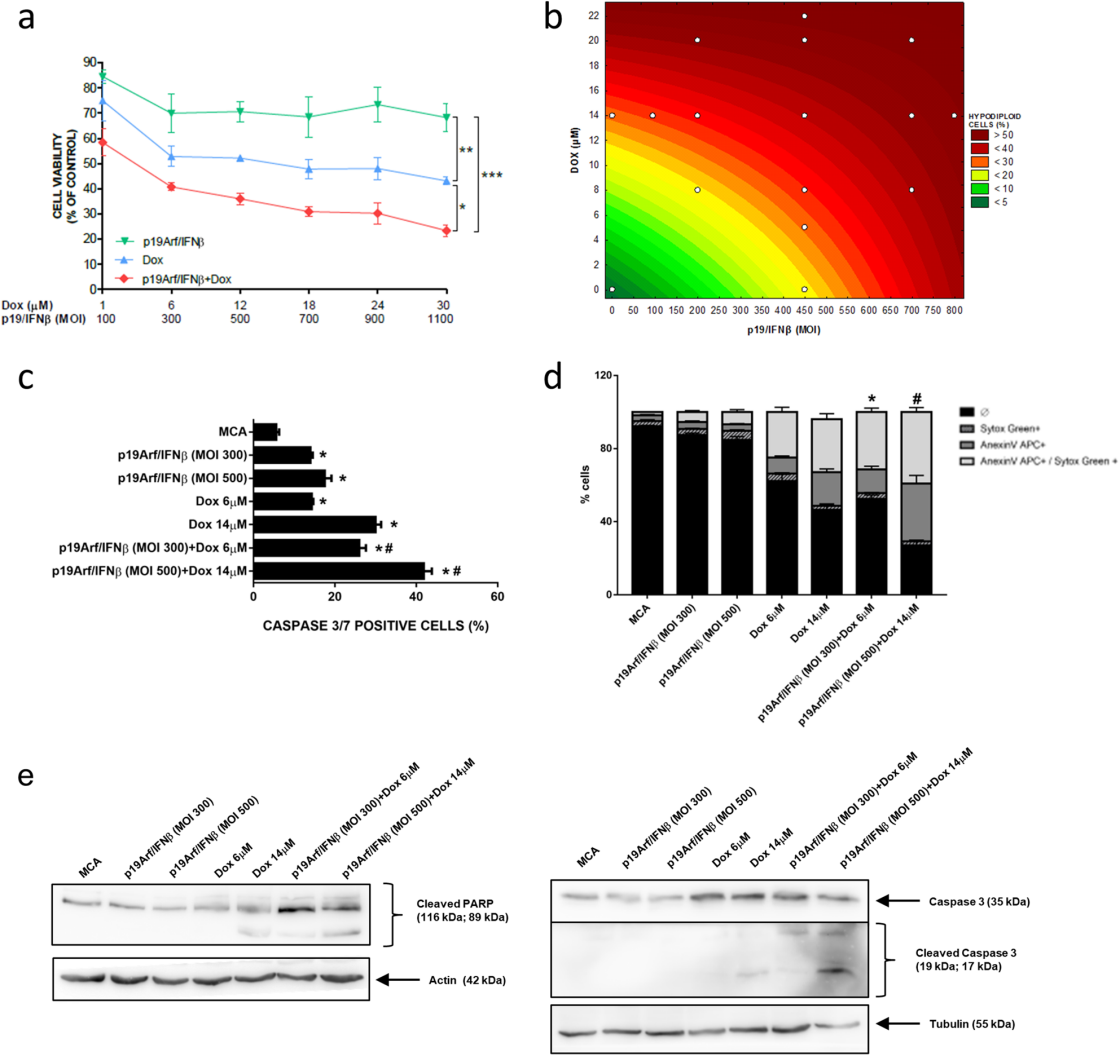
In vitro association of p19Arf/IFNβ and doxorubicin augments cell death levels. (**a**) MTT analysis of MCA cells treated for 12 h individually or in combination with doxorubicin and AdRGD-PG-p19 and AdRGD-PG-IFNβ vectors. n = 4 [One-way Anova and Tukey’s multiple comparison post-test]. (**b**) Response surface plot illustrating the influence of different p19Arf/IFNβ MOIs and doxorubicin concentrations on the percentage of hypodiploid MCA cells after 20 h of treatment, for which interactions were considered significant by the ANOVA and Pareto chart analysis. (**c**) Flow cytometry analysis of caspase 3 activity in MCA cells after 16 h of p19Arf/IFNβ and of doxorubicin treatment. Each assay performed on 3 independent occasions with technical duplicates. One-way Anova with Sidak’s post-test. *p < 0.0001 vs CTR; ^#^p < 0.0001 p19Arf/IFNβ + Dox vs p19Arf/IFNβ or Dox (**d**) Fresh MCA cells, treated as indicated, were stained to reveal apoptotic cells (AnnexinV APC +) or advanced cell death (Sytox Green +) before observation by flow cytometry. Each assay performed on 3 independent occasions with technical duplicates. Two-way Anova with Tukey’s post-test. (**e**) Representative image of two independent Western blotting experiments of cleaved PARP or caspase 3 with or without cleavage upon treatment of MCA cells (N = 3).

**Figure 3:**
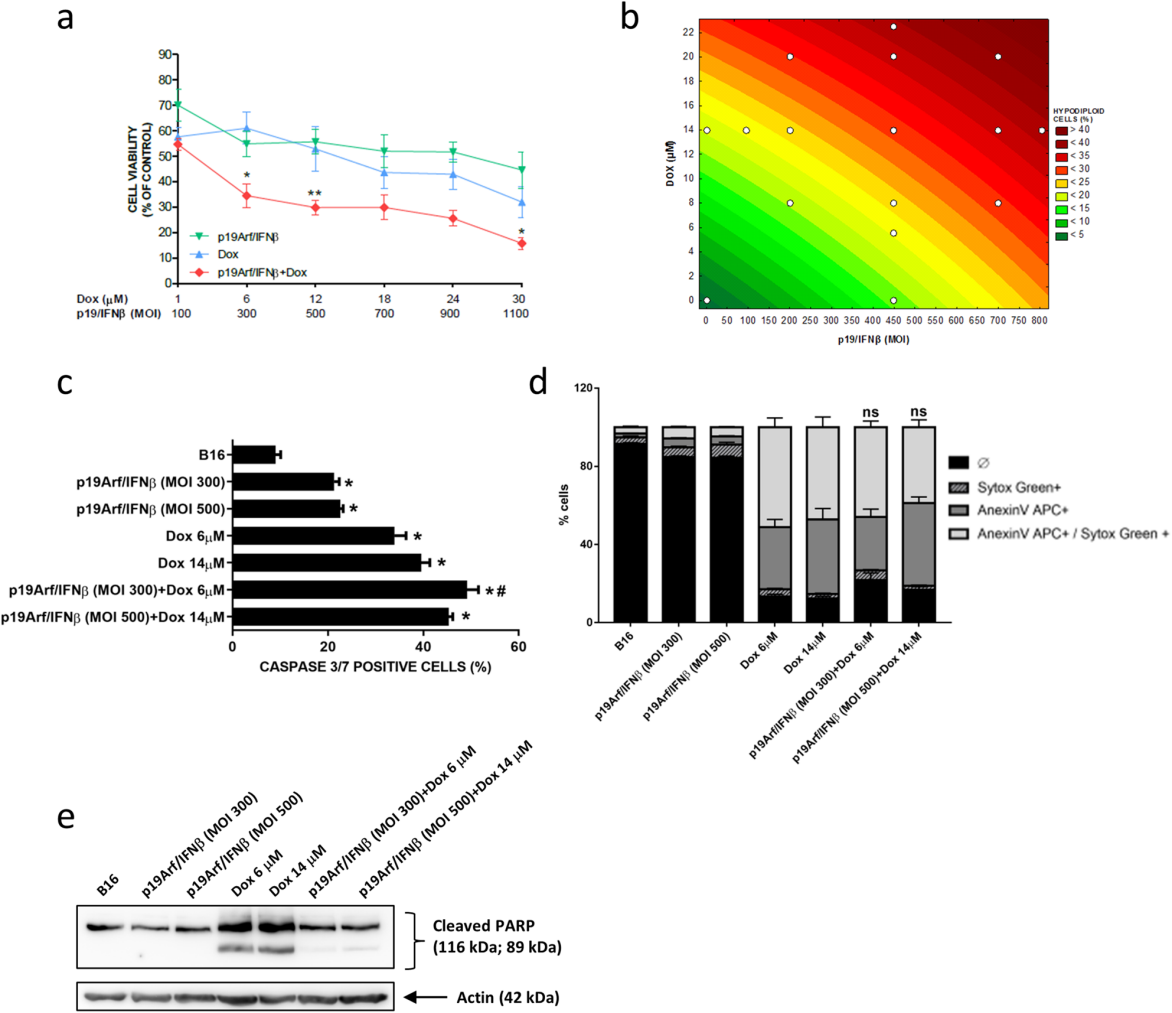
In vitro association of p19Arf/IFNβ and doxorubicin augments cell death levels. (**a**) MTT analysis of B16 cells treated for 12 h individually or in combination with doxorubicin and AdRGD-PG-p19 and AdRGD-PG-IFNβ vectors. n = 4 [One-way Anova and Tukey’s multiple comparison post-test]. (**b**) Response surface plot illustrating the influence of different p19Arf/IFNβ MOIs and doxorubicin concentrations on the percentage of hypodiploid MCA cells after 20 h of treatment, for which interactions were considered significant by the ANOVA and Pareto chart analysis. (**c**) Flow cytometry analysis of caspase 3 activity in B16 cells after 16 h of p19Arf/IFNβ and of doxorubicin treatment. Each assay performed on 3 independent occasions with technical duplicates. One-way Anova with Sidak’s post-test. *p < 0.0001 vs CTR; ^#^p < 0.0001 p19Arf/IFNβ + Dox vs p19Arf/IFNβ or Dox. (**d**) Fresh B16 cells, treated as indicated, were stained to reveal apoptotic cells (AnnexinV APC +) or advanced cell death (Sytox Green +) before observation by flow cytometry. Each assay performed on 3 independent occasions with technical duplicates. Two-way Anova with Tukey’s post-test. (**e**) Representative image of two independent Western blot experiments for detection of cleaved PARP upon treatment of B16 cells.

We expect that the association between p19Arf/IFNβ and Dox would act synergistically in the induction of cell death, thus allowing a reduction in both virus MOI and Dox concentration. To test this hypothesis, cell viability of MCA and B16 cells was analyzed after exposure to increasing doses of Dox (1–30 µM) as well as virus particles (MOI 100–1100), applied in association or individually. A dramatic reduction in cell viability was observed in either cell line where combined treatment surpassed the effect caused by monotherapies, as noted by the lower doses of the combination (MOI 300 with 6 µM of Dox) in contrast to the higher doses employed for the single agents (MOI 1100 or 30 µM of Dox, Figs. 2a and 3a). To further support this finding, we next performed central composite rotational design (CCRD) analysis by combining different Dox concentrations and viral MOIs and evaluated the impact on promoting cell death. MCA and B16 were treated with different combinations of chemotherapy and p19Arf/IFNβ (detailed in Tables S1 and S2) and the percentage of hypodiploid cells projected in a surface response graph (Figs. 2b and 3b). Through this analysis, the association of p19Arf/IFNβ and Dox was shown to significantly enrich the induction of cell death, even in conditions where Dox concentration and virus MOI were decreased in an interchangeable manner, indicating that by sensitizing cells to Dox chemotherapy, p19Arf/IFNβ allows use of lower doses with potentially less severe side effects. Furthermore, we investigated the activity of caspase 3, known for playing a central role in the execution-phase of apoptosis^28^ yet, based on our previous study, is not involved in the necroptotic cell death induced by p19Arf/IFNβ^14^. Accordingly, in both cell lines, treatment with just p19Arf/IFNβ provided relatively low caspase 3/7 activity, in sharp contrast to 14 µM Dox, where activity was seen in about 30% of cells (Figs. 2c and 3c). In the MCA cell line, p19Arf/IFNβ + Dox treatment induced caspase 3/7 activity in more than 40% of cells, suggesting an additive effect that may explain why this association strongly induces cell death (Fig. 2c). Additional indicators of cell death, annexinV staining and cleavage of PARP and caspase 3, corroborate these findings for MCA (Fig. 2d,e). In B16 cells, the additive effect of p19Arf/IFNβ + Dox treatment on caspase 3/7 activity is more subtle (Fig. 3c), and is not reflected in annexinV staining or cleavage of PARP (Fig. 3d,e), possibly related to the induction of necroptosis, not apoptosis, as noted in our previous work with this cell line^14^.

### Impact of p19Arf/IFNβ and Dox association on tumor immunogenicity

**Figure 4:**
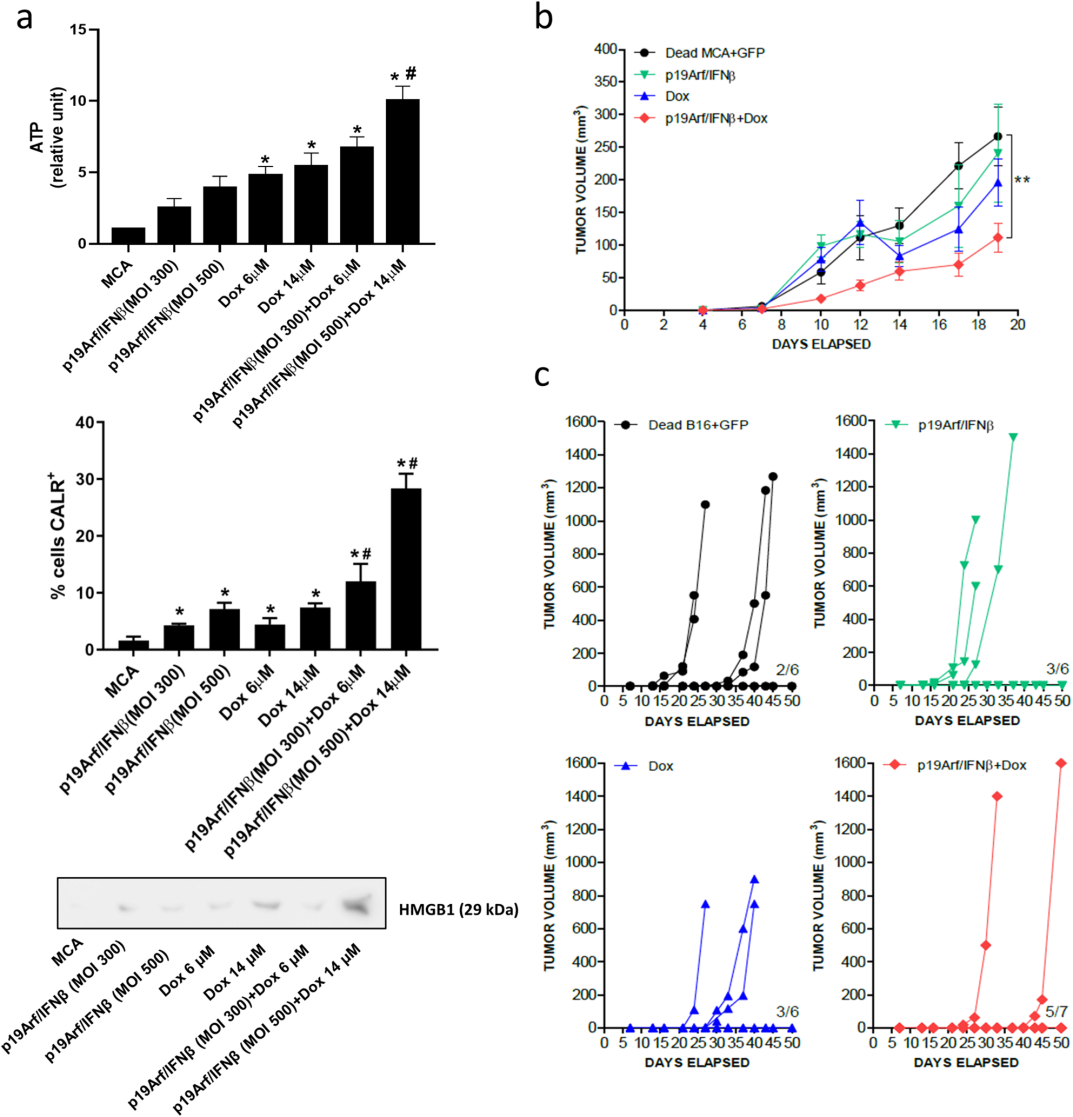
p19Arf/IFNβ and doxorubicin association potentiates immunogenicity of treated cancer cells. (**a**) Detection of ICD-related DAMPS in the MCA cell line: ATP (secreted, detected by ELISA), calreticulin (CALR, cell surface detection by flow cytometry) or HMGB1 (secreted, detected by western blot of cell concentrated cell supernatant). For ATP and CALR, each assay performed on 3 independent occasions with technical duplicates. One-way Anova with Sidak’s post-test. *p < 0.0001 vs CTR; ^#^p < 0.0001 p19Arf/IFNβ + Dox vs p19Arf/IFNβ or Dox The western blot was performed on 3 independent occasions; a representative image is presented. (**b**) MCA tumors were established (s.c.) before immunotherapy was performed using MCA cells treated ex vivo as indicated. Impact of immunotherapy on progression of challenge MCA tumors was monitored. [Two-way Anova and Bonferroni post-test] n = 6 for all groups, except for p19Arf/IFNβ + Dox where n = 7. **p < 0.001 p19Arf/IFNβ + Dox vs Dead MCA + GFP. (**c**) B16 tumors (s.c.) were established then immunotherapy performed using B16 cells treated ex vivo as indicated. Progression of the s.c. (challenge) tumors were monitored.

A possible benefit of the p19Arf/IFNβ and Dox association would be to potentiate the antitumor immune response unleashed by each of these therapies. We hypothesize that by using two distinct ICD inducers, secretion of ICD DAMPS could be modulated and result in increased immunogenicity of the treated cells^13^. To address this hypothesis, we examined key markers of ICD in response to the p19Arf/IFNβ + Dox treatment. While either therapy alone could induce emission of the ICD markers in MCA cells, the association of p19Arf/IFNβ + Dox (MOI 500 + 14 µM) provided the highest intensity (Fig. 4a).

To test if the p19Arf/IFNβ + Dox treatment results in superior immunogenicity of the treated cells, we employed a cancer immunotherapy model in which MCA or B16 cells were treated ex vivo with p19Arf/IFNβ, Dox or their combination, and before the start of the cell death process (i.e., not detectable by PI staining), cells were injected (s.c) to die within the host and function as a cancer vaccine immunogen against a previously established growing tumor, termed as challenge tumor. Remarkably, only vaccination with p19Arf/IFNβ + Dox MCA cells reduced progression of challenge tumors, whereas cells treated with just p19Arf/IFNβ or Dox displayed little protective effect when compared to mice that received AdRGD-PG-eGFP transduced cells killed by freeze and thaw, a control expected to induce accidental necrosis and tumor antigen release **(**Fig. 4b). However, it is important to note that mice vaccinated with cells treated with just p19Arf/IFNβ developed tumors at the vaccine site at late time points (Fig. S2), which may be due to resistant clones already present in this cell line that, in vitro, were observed to repopulate the tissue plate after p19Arf/IFNβ therapy (data not shown). Therapeutic vaccination was also performed with B16 cells and once again p19Arf/IFNβ + Dox B16 cells profoundly inhibited progression of challenge tumors, as evidenced by 5 out 7 mice that fully rejected their tumors (Fig. 4c) and consequently presented survival superior to the monotherapy groups (Fig. S3). Although the immunological mechanism involved in this antitumor response needs to be investigated in more detail, these results provide strong evidence for the ability of the p19Arf/IFNβ + Dox association to augment immunogenicity of treated cells.

### In situ association of p19Arf/IFNβ and Dox

**Figure 5:**
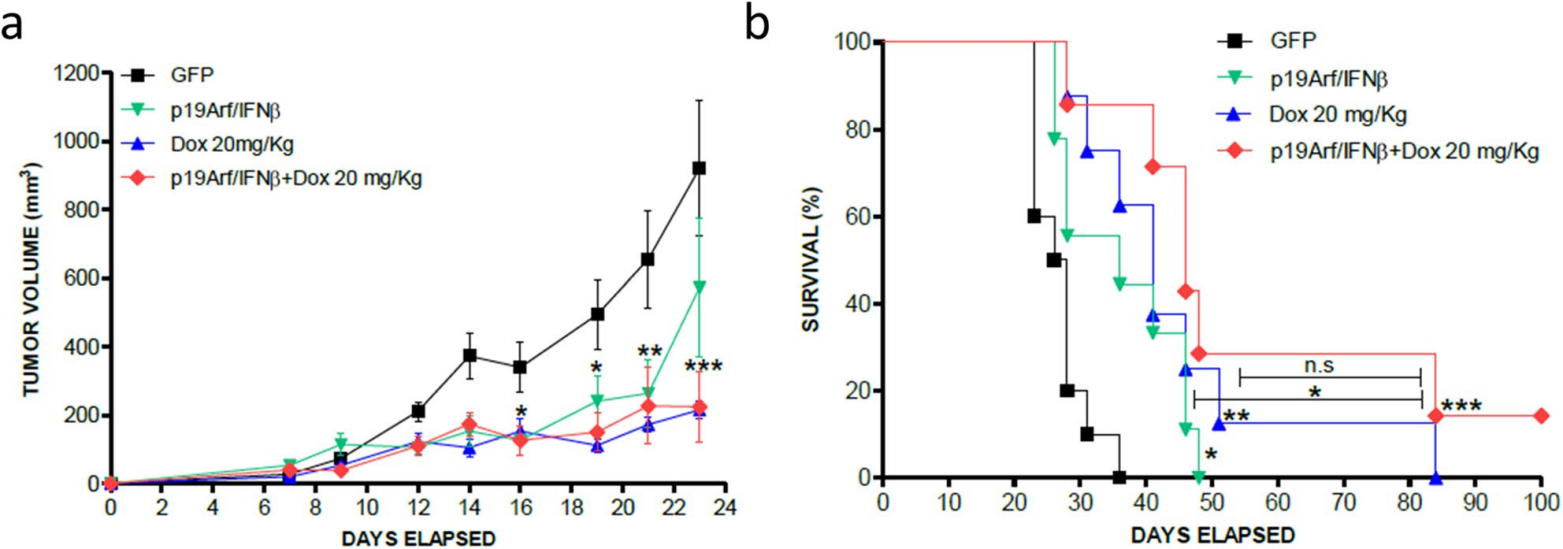
In vivo association of p19Arf/IFNβ gene therapy with doxorubicin inhibits tumor progression and changes the dynamic of caspase 3 activity. (**a**) Tumor progression curves of established MCA-DEVD tumors that received in situ gene therapy with the AdRGD-PG-p19 and AdRGD-PG-IFNβ vectors and after 48 h, treated with 20 mg/kg of doxorubicin as indicated. [Two-way Anova and Bonferroni post-test] n = 6 for all groups. (**b**) Survival analysis of mice from **a**. [Log Rank Mantel-cox test, followed by Wilcoxon post- Test. n = 8 for all groups, except p19Arf/IFNβ which n = 9.

Next, we sought to investigate the therapeutic impact of associating in situ p19Arf/IFNβ with Dox in comparison with the monotherapies. To this end, established MCA-DEVD tumors, which were stably modified to expresses a caspase 3 reporter, were treated in situ with three rounds of AdRGD-PG-p19 and AdRGD-PG-IFNβ gene therapy and two days later, also injected with the therapeutic dose of Dox, 20 mg/kg. From this assay, we noticed that individual treatments with p19Arf/IFNβ and Dox similarly reduced tumor progression when compared to the GFP control treatment (Fig. 5a). However, as the tumor progresses, this similarity is lost, since tumors treated with just p19Arf/IFNβ begin to grow more than Dox-treated tumors. The combined use of these treatments was strikingly effective not only in decreasing tumor volume, but also in conferring a survival benefit (Fig. 5b). Interestingly, only in the association group, complete regression was observed in one mouse, evidenced by reduced volume shortly after treatment, followed by a 40 day period of stable volume, then regressed completely by day 70, a result not demonstrated in the graph of tumor volume, but contemplated in the survival curve. In this way, treatment with Dox and its association with p19Arf/IFNβ were the most effective modalities for increasing survival.

Moreover, through the analysis of caspase 3 activity in a bioluminescence imaging strategy, treatment with Dox alone or in association with p19Arf/IFNβ in situ resulted in elevated luciferase reporter activity, thus indicating increased caspase 3 activity (Fig. S4). In contrast, neither the treatment with the GFP or p19Arf/IFNβ vectors resulted in increased caspase 3 activity. This assay suggests that pretreatment with p19Arf/IFNβ, just as in vitro, does not activate caspase 3. However, as compared to Dox treatment, this assay did not reveal potentiation of caspase 3 when p19Arf/IFNβ was associated with Dox.

**Figure 6:**
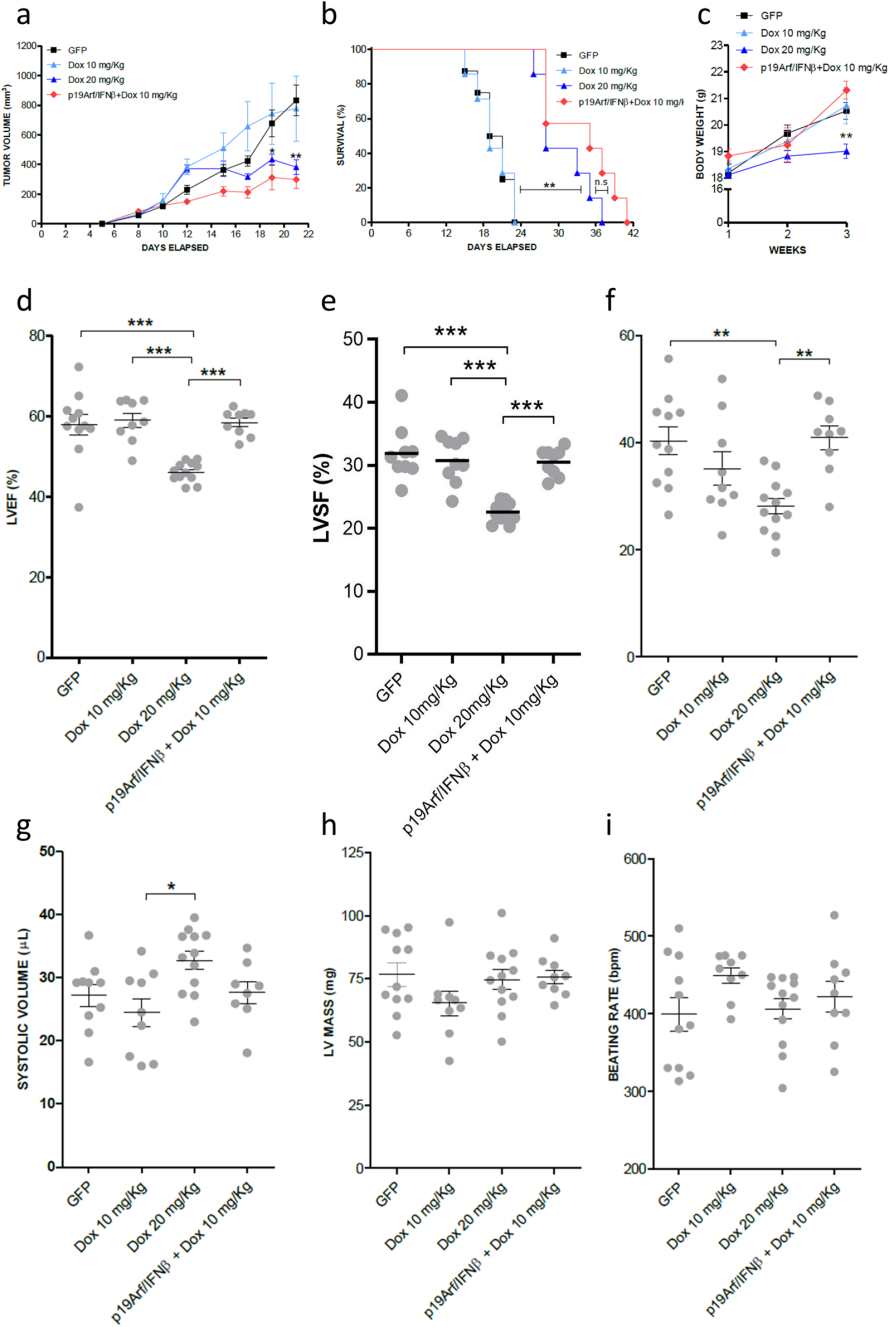
Pre-treatment with p19Arf/IFNβ gene therapy restores efficacy of a sub-therapeutic dose of doxorubicin yet maintains cardiac function. (**a**) Tumor progression curves of established MCA tumors that were treated with 10 or 20 mg/kg of doxorubicin or in the association group, treated with the AdRGD-PG-p19 and AdRGD-PG-IFNβ in situ gene therapy and after 48 h, also treated with 10 mg/kg of doxorubicin. [Two-way Anova and Bonferroni post-test] n = 8 for all groups. (**b**) Survival analysis of mice from (**a**). [Log Rank Mantel-cox test, followed by Wilcoxon post-test]. (**c**) Monitoring of body weight during therapy [Two-way Anova and Bonferroni post-test]. Echocardiogram analysis of (**d**) ejection fraction, (**e**) left ventricular systolic function, (**f**) stroke volume, (**g**) systolic volume, (**h**) left ventricular (LV) mass and (**i**) beating rate of heart 10 days after therapy. n = 8 for each group. [One-way Anova, followed by Tukey’s multiple comparison post-test].

Having observed that the association between p19Arf/IFNβ gene transfer with the therapeutic dose of Dox does not further reduce tumor volume as compared to Dox alone, we next asked whether the benefit of this association would be maintained when using a sub-therapeutic dose of Dox, 10 mg/kg, and consequently ease induction of cardiotoxicity, a major side effect observed in the clinical setting. Therefore, after in situ treatment with AdRGD-PG-p19 and AdRGD-PG-IFNβ gene therapy, MCA tumors were injected (i.t) with 10 mg/kg of Dox, which when used individually does not reduce tumor progression to the same extent as the dose of 20 mg/kg (Fig. 6a). In accordance with our hypothesis and in vitro data, treatment with p19Arf/IFNβ elevated the efficacy of the 10 mg/kg dose of Dox to the same level as the dose of 20 mg/kg, providing significant reduction in tumor progression as well as substantial increase in survival (Fig. 6b), when compared to control groups GFP + PBS and Dox 10 mg/kg as monotherapy. However, those mice in the Dox 20 mg/kg group failed to gain weight during the three weeks of therapy (Fig. 6c) and even more critically, as analyzed by echocardiogram, a profound impairment of cardiac function was revealed in these animals. Indeed, we revealed compromised left ventricular ejection function (LVEF) and left ventricular systolic function (LVSF) parameters (Fig. 6d–g). We also observed that ventricular mass was not altered by the treatment, which indicates that no aggravating anatomic changes were detected up to the moment of the analysis (Fig. 6h). Moreover, through the beating rate, we can attest that this analysis was performed following a standardized protocol, as mice displayed similar beating rates per minute (Fig. 6i). It is important to note that this analysis was performed 10 days after the i.t. treatment of Dox, representing an acute post-therapy event. This assay shows that the association of p19Arf/IFNβ + Dox permitted the use of a lower drug dosage, resulting in both preserved cardiac function as well as effective inhibition of tumor progression.

## Discussion

In this study we have explored potential benefits of applying p19Arf/IFNβ gene therapy in association with Dox chemotherapy, both of which elicit ICD, and as postulated here, their interplay could act on different levels of the therapeutic response. Previously, we have shown that reintroduction of p19Arf to cancer cells that harbor wild type p53 leads to the activation of the p53 apoptotic program, but when an IFNβ antiviral context is induced simultaneously, an alternative mechanism of cell death is unleashed acting in a caspase 3 independent manner and through the up-regulation of RIP3K, a critical mediator of necroptosis^14^. Here, the association of p19Arf/IFNβ + Dox resulted in increased levels of cell death accompanied by increased caspase 3 activity, permitting the reduction in virus and drug doses, resulting in the effective control of tumor progression while avoiding cardiotoxicity caused by higher doses of Dox.

Dox and Nutlin-3 are well known for their ability to activate p53, a point we demonstrate here and in our previous work^29^. Interestingly, these treatments resulted in increased reporter gene activity, implying that the MCA cell line is endowed with transcriptionally functional p53 that can regulate expression from the PG promoter. Although not experimentally demonstrated here, activation of p53 by the combined use of these agents may further stimulate the PG promoter and augment transgene expression levels, a point addressed experimentally in our previous work^30^. In our approach, treatment with the AdRGD-PG-p19 and AdRGD-PG-IFNβ vectors as well as Dox are all expected to activate p53, resulting in enhanced expression from the p53-responsive vectors as well as a multi-pronged approach to the induction of cell death.

Indeed, Dox is a well-known inducer of apoptosis and caspase 3/7 activity^11^, as seen in this work when applied alone or in conjunction with p19Arf/IFNβ gene transfer. This was shown through the analysis of caspase 3 in vivo, where Dox alone or in combination with p19Arf/IFNβ resulted in prolonged caspase 3-dependent luciferase activity when comparing the 48-h vs. 24-h time points. This suggests that in vivo the cell death pathway induced by p19Arf/IFNβ treatment was modulated by the addition of Dox, as observed in our in vitro experiments. CCRD analysis suggests that the viral MOI and drug concentration may be reduced since these treatments act cooperatively. Thus, the dynamics of vector expression, transgene activity and chemotherapy are acting in concert to bring about cell death.

Although massive cell death may be needed to reduce tumor volume, induction of ICD can also evoke antitumor immunity. Therefore, combinatorial use of distinct agents could provide additive stimuli, modulating the extent of cell death as well as the immunogenicity of treated cells. As shown here, ex vivo treatment of cells with p19Arf/IFNβ + Dox provided superior antitumor protection in a therapeutic vaccine setting, a result that presumably depends on the up-regulation of some of the known ICD mediators, as shown here. Even so, additional factors may be in play. Modulation of the immunogenic potential of cancer cells has already been demonstrated with cisplatin (CDDP) that as a single agent does not promote endoplasmic reticulum stress and therefore no translocation of calreticulin to the cell surface^31^. But the association with thapsigargin, an inhibitor of the sarco/ER Ca(2+)-ATPase, endows CDDP with this ability and therefore a role in ICD. Thus, in future studies it will be interesting to investigate which of the ICD mediators are actually critical or even if high levels IFNβ provided by our vector can circumvent the lack of expression of one of them—a matter with important implications for cancer patients that present dysfunction in the ability to succumb to *bona fide* ICD, as already identified in breast cancer patients who carry a toll like receptor 4 (TLR4) loss-of-function allele and consequently a defect in HMGB1 binding^10^.

Additionally, modulation of immunogenicity should impact how dying cells interact with different APC subsets. For example, CD169 + macrophages have been shown to dominate antitumor immunity by cross-presenting dead cell-associated antigens^32^. Expression of our AdRGD-PG-IFNβ vector should also bring about the strong maturation and differentiation stimuli that are associated with IFNβ, possibly impacting tumor associated DCs. In particular, it would be interesting to study the role of Baft3 DCs which, upon activation of the STING-IFNβ pathway, can mediate recruitment of T cells within the TME through the CXCL9/CXCL10 axis^33,34^.

Yet, infliction of cell death can also negatively impact therapeutic outcome, as observed with localized radiotherapy that mediates caspase 3 activation and regulates prostaglandin E2 production_,_ stimulating growth of surviving tumor cells and favoring tumor repopulation^24^. Moreover, capture of apoptotic cell by CD169^+^ macrophages has also been implicated in promoting rapid expression of the chemokine CCL22, inducing migration of FoxP3^+^ Tregs to the spleen and their activation, favoring classic apoptotic cell-induced immune suppression^35^. Along these lines, induction of inhibitory immune checkpoints could also come into play and hamper immunity. It is tempting to speculate that these mechanisms may explain why the therapeutic effect from the association of p19Arf/IFNβ with the 20 mg/kg was not more pronounced as compared to their application as monotherapies. It will be critical to evaluate levels of PD-L1 expression on tumor associated CD45 + and CD45- cells upon p19Arf/IFNβ gene therapy, which besides revealing if cancer cells or immune host cells are actually mediating immune resistance, may also provide rational for associating PD-1 checkpoint blockade, an immunotherapy proposed as the cornerstone for most combinatorial immunotherapeutic strategies^36^, especially for those that rely on promoting strong inflammatory immune responses.

The relationship between intratumoral doses of Dox and their immunomodulatory properties has been reported previously^27,37–41^. The evidence indicates that the efficacy seen at each dose may be influenced not only by the tumor cell type, but also by the tumor size^42^ or timing when treatment is applied, resulting in dynamic changes in the immune infiltrate that correlate with tumor progression, including accumulation of myeloid cells and a reduction in the number of functional effector T cells^43,44^. Even though the 5 and 10 mg/kg doses were considered as sub-therapeutic, the outcome may have been quite different if applied earlier during progression or in other cancer types. But then again, we believe that our model, where fully established tumors are treated, may better reflect the advanced stage at which most cancer patients are enrolled into the clinic. And it is in this scenario that association of an immunotherapy capable of promoting cell death and immune stimulation, such as ours, may offer an advantage over other strict immune modulators, specifically, sensitizing cancer cells to cell death and modulating the surrounding TME with respect to immune function. Future studies will be required to explore the full extent of the immune stimulation in response to the p19Arf/IFNβ + Dox associated therapy.

While immune activation was not thoroughly explored here, the interactions between p19Arf/IFNβ and Dox were shown to significantly potentiate induction of cell death, allowing the use of smaller doses of Dox as well reduced virus MOI**.** Accordingly, association with p19Arf/IFNβ with a subtherapeutic dose of Dox, 10 mg/kg, resulted in the effective inhibition of tumor progression, yet preserved cardiac function. While higher dosages of Dox could control tumor progression, acute cardiotoxicity was encountered. Thus, the association of p19Arf/IFNβ + low dose Dox was advantageous.

In conclusion, evidence presented here unveiled relevant therapeutic benefits of using the p19Arf and IFNβ gene therapy in association with Dox, a standard of care immunogenic chemotherapeutic agent. This study paves the way for the exploration of combinatorial approaches, such as gene transfer in association with other inducers of immunogenic cell death or immune stimulatory modalities, harboring great potential for the further improvement of therapeutic efficacy while avoiding cardiotoxicity.

## Supplementary Information

Supplementary Information 1.

Supplementary Information 2.

## References

[1] Kaneno R, Shurin GV, Tourkova IL, Shurin MR (2009). Chemomodulation of human dendritic cell function by antineoplastic agents in low noncytotoxic concentrations. J. Transl. Med..

[2] Arlen PM, Madan RA, Hodge JW, Schlom J, Gulley JL (2007). Combining vaccines with conventional therapies for cancer. Update Cancer Ther..

[3] Ding ZC, Blazar BR, Mellor AL, Munn DH, Zhou G (2010). Chemotherapy rescues tumor-driven aberrant CD4+ T-cell differentiation and restores an activated polyfunctional helper phenotype. Blood.

[4] Zhang L (2008). Differential impairment of regulatory T cells rather than effector T cells by paclitaxel-based chemotherapy. Clin. Immunol..

[5] Le HK (2009). Gemcitabine directly inhibits myeloid derived suppressor cells in BALB/c mice bearing 4T1 mammary carcinoma and augments expansion of T cells from tumor-bearing mice. Int. Immunopharmacol..

[6] Galluzzi L (2020). Consensus guidelines for the definition, detection and interpretation of immunogenic cell death. J. Immunother. Cancer.

[7] Kroemer G, Galluzzi L, Kepp O, Zitvogel L (2013). Immunogenic cell death in cancer therapy. Annu. Rev. Immunol..

[8] Michaud M (2011). Autophagy-dependent anticancer immune responses induced by chemotherapeutic agents in mice. Science.

[9] Obeid M (2007). Calreticulin exposure dictates the immunogenicity of cancer cell death. Nat. Med..

[10] Apetoh L (2007). Toll-like receptor 4-dependent contribution of the immune system to anticancer chemotherapy and radiotherapy. Nat. Med..

[11] Casares N (2005). Caspase-dependent immunogenicity of doxorubicin-induced tumor cell death. J. Exp. Med..

[12] Garg AD, Krysko DV, Vandenabeele P, Agostinis P (2012). Hypericin-based photodynamic therapy induces surface exposure of damage-associated molecular patterns like HSP70 and calreticulin. Cancer Immunol. Immunother.

[13] Galluzzi L, Buque A, Kepp O, Zitvogel L, Kroemer G (2017). Immunogenic cell death in cancer and infectious disease. Nat. Rev. Immunol..

[14] Hunger A (2017). Reestablishment of p53/Arf and interferon-β pathways mediated by a novel adenoviral vector potentiates antiviral response and immunogenic cell death. Cell Death Discov..

[15] Medrano RFV, Hunger A, Catani JPP, Strauss BE (2017). Uncovering the immunotherapeutic cycle initiated by p19Arf and interferon-β gene transfer to cancer cells: An inducer of immunogenic cell death. Oncoimmunology.

[16] Merkel CA, Medrano RF, Barauna VG, Strauss BE (2013). Combined p19Arf and interferon-beta gene transfer enhances cell death of B16 melanoma in vitro and in vivo. Cancer Gene Ther..

[17] Medrano RF (2016). Vaccination using melanoma cells treated with p19arf and interferon beta gene transfer in a mouse model: A novel combination for cancer immunotherapy. Cancer Immunol. Immunother..

[18] Catani JP (2016). Intratumoral Immunization by p19Arf and Interferon-β Gene Transfer in a Heterotopic Mouse Model of Lung Carcinoma. Transl. Oncol..

[19] Thorn CF (2011). Doxorubicin pathways: Pharmacodynamics and adverse effects. Pharmacogenet. Genomics.

[20] Bajgelman, M. C. M., R.F.V. Carvalho, A.C.P.V. Strauss, B.E.

[21] Octavia Y (2012). Doxorubicin-induced cardiomyopathy: from molecular mechanisms to therapeutic strategies. J. Mol. Cell Cardiol..

[22] Angsutararux P, Luanpitpong S, Issaragrisil S (2015). Chemotherapy-induced cardiotoxicity: Overview of the roles of oxidative stress. Oxid. Med. Cell Longev..

[23] Fridlender ZG (2010). Chemotherapy delivered after viral immunogene therapy augments antitumor efficacy via multiple immune-mediated mechanisms. Mol. Ther..

[24] Huang Q (2011). Caspase 3-mediated stimulation of tumor cell repopulation during cancer radiotherapy. Nat. Med..

[25] Tamura RE, Silva Soares RB, Costanzi-Strauss E, Strauss BE (2016). Autoregulated expression of p53 from an adenoviral vector confers superior tumor inhibition in a model of prostate carcinoma gene therapy. Cancer Biol. Ther..

[26] Lang RM (2005). Recommendations for chamber quantification: a report from the American Society of Echocardiography's Guidelines and Standards Committee and the Chamber Quantification Writing Group, developed in conjunction with the European Association of Echocardiography, a branch of the European Society of Cardiology. J. Am. Soc. Echocardiogr..

[27] Ma Y (2011). Contribution of IL-17-producing gamma delta T cells to the efficacy of anticancer chemotherapy. J. Exp. Med..

[28] Galluzzi L (2012). Molecular definitions of cell death subroutines: recommendations of the Nomenclature Committee on Cell Death 2012. Cell Death Differ.

[29] Bajgelman MC, Medrano RF, Carvalho AC, Strauss BE (2013). AAVPG: A vigilant vector where transgene expression is induced by p53. Virology.

[30] Merkel CA (2010). Activation of endogenous p53 by combined p19Arf gene transfer and nutlin-3 drug treatment modalities in the murine cell lines B16 and C6. BMC Cancer.

[31] Martins I (2011). Restoration of the immunogenicity of cisplatin-induced cancer cell death by endoplasmic reticulum stress. Oncogene.

[32] Asano K (2011). CD169-positive macrophages dominate antitumor immunity by crosspresenting dead cell-associated antigens. Immunity.

[33] Spranger S, Dai D, Horton B, Gajewski TF (2017). Tumor-residing Batf3 dendritic cells are required for effector T cell trafficking and adoptive T cell therapy. Cancer Cell.

[34] Corrales L, McWhirter SM, Dubensky TW, Gajewski TF (2016). The host STING pathway at the interface of cancer and immunity. J. Clin. Invest..

[35] Ravishankar B (2014). Marginal zone CD169+ macrophages coordinate apoptotic cell-driven cellular recruitment and tolerance. Proc. Natl. Acad. Sci. U S A.

[36] Melero I (2015). Evolving synergistic combinations of targeted immunotherapies to combat cancer. Nat. Rev. Cancer.

[37] Ma Y (2013). Tumor necrosis factor is dispensable for the success of immunogenic anticancer chemotherapy. Oncoimmunology.

[38] Viaud S (2013). The intestinal microbiota modulates the anticancer immune effects of cyclophosphamide. Science.

[39] Ma Y (2013). Anticancer chemotherapy-induced intratumoral recruitment and differentiation of antigen-presenting cells. Immunity.

[40] Vacchelli E (2015). Chemotherapy-induced antitumor immunity requires formyl peptide receptor 1. Science.

[41] Xu M (2015). Intratumoral delivery of IL-21 overcomes Anti-Her2/Neu resistance through shifting tumor-associated macrophages from M2 to M1 phenotype. J. Immunol..

[42] Wen FT, Thisted RA, Rowley DA, Schreiber H (2012). A systematic analysis of experimental immunotherapies on tumors differing in size and duration of growth. Oncoimmunology.

[43] Vilain RE (2017). Dynamic changes in PD-L1 expression and immune infiltrates early during treatment predict response to PD-1 blockade in melanoma. Clin. Cancer Res..

[44] Lavin Y (2017). Innate immune landscape in early lung adenocarcinoma by paired single-cell analyses. Cell.

