# Supplementary material for: Potentiation of combined p19Arf and interferon-beta cancer gene therapy through its association with doxorubicin chemotherapy
Source: Sci Rep. 2022 Aug 10;12:13636. doi: 10.1038/s41598-022-17775-y (PMC9365852; doi:10.1038/s41598-022-17775-y)
Supplement: Supplementary file 1 — Supplementary Information 1. [file 41598_2022_17775_MOESM1_ESM.pdf]

Table S1: Variables and levels of the Central Composite Rotational Design (CCRD) assay

	Levels					
	**(- α)**	**Inferior**	**Central**	**Superior**	**(+ α)**	
Variables	-1.414	-1	0	1	+1.414	step
X1<br>p19Arf/IFN-β<br>(MOI)	196.5	300	550	800	903.5	250
X2<br>Dox (μM)	5.516	8	14	20	22.48	6

Table S2: Experimental factorial design used to evaluate the percentage of hypodiploid cells after combinatory treatment with adenovirus and/or doxorubicin (Dox).

Condition	X1 (Vector)	X2 (Dox)	p19/IFN-β (MOI)	Dox (µM)	
**1**	(-) 1	(-) 1	300	8	**Central Composite Rotational Design (CCRD)**
**2**	(+) 1	(-) 1	800	8	
**3**	(-) 1	(+) 1	300	20	
**4**	(+) 1	(+) 1	800	20	
**5**	(-) 1	(0) Central	300	14	
**6**	(+) 1	(0) Central	800	14	
**7**	(0) Central	(-) 1	550	8	
**8**	(0) Central	(+) 1	550	20	
**9**	(0) Central	(-) 1.414	550	5.516	**CCRD Rotatation**
**10**	(0) Central	(+) 1.414	550	22.48	
**11**	(-) 1.414	(0) Central	196.5	14	
**12**	(+) 1.414	(0) Central	903.5	14	
**13**	(0) Central	(0) Central	550	14	**CP Central points**
**14**	(0) Central	(0) Central	550	14	
**15**	(0) Central	(0) Central	550	14	
**16**	(0) Central	(0) Central	550	14	
**17**	(0) Central	(0) Central	550	14	
**18**	Untreated	Untreated	0	0	**C Controls**
**19**	(0) Central	Untreated	550	0	
**20**	Untreated	(0) Central	0	14	

Table S2

a

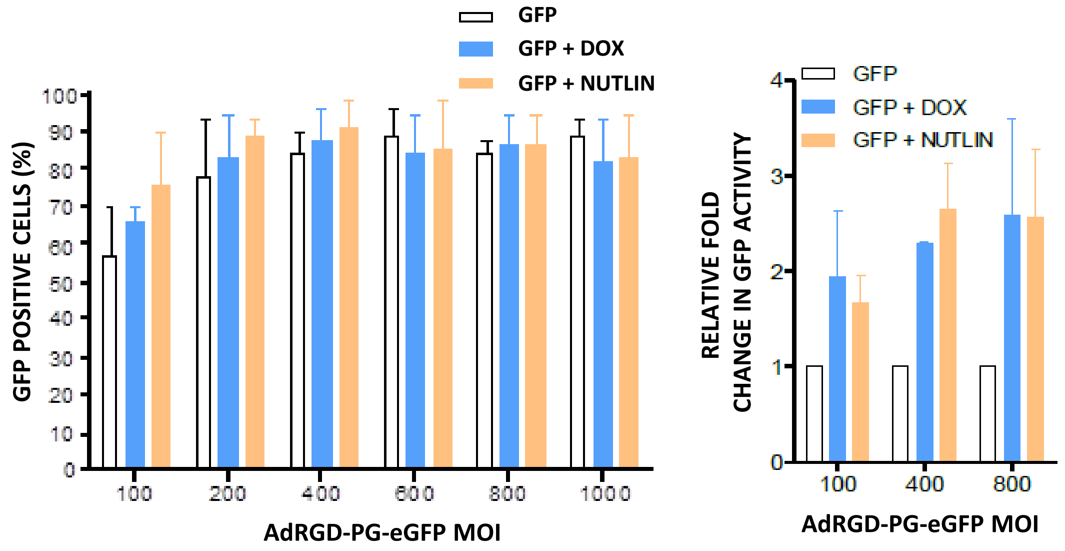

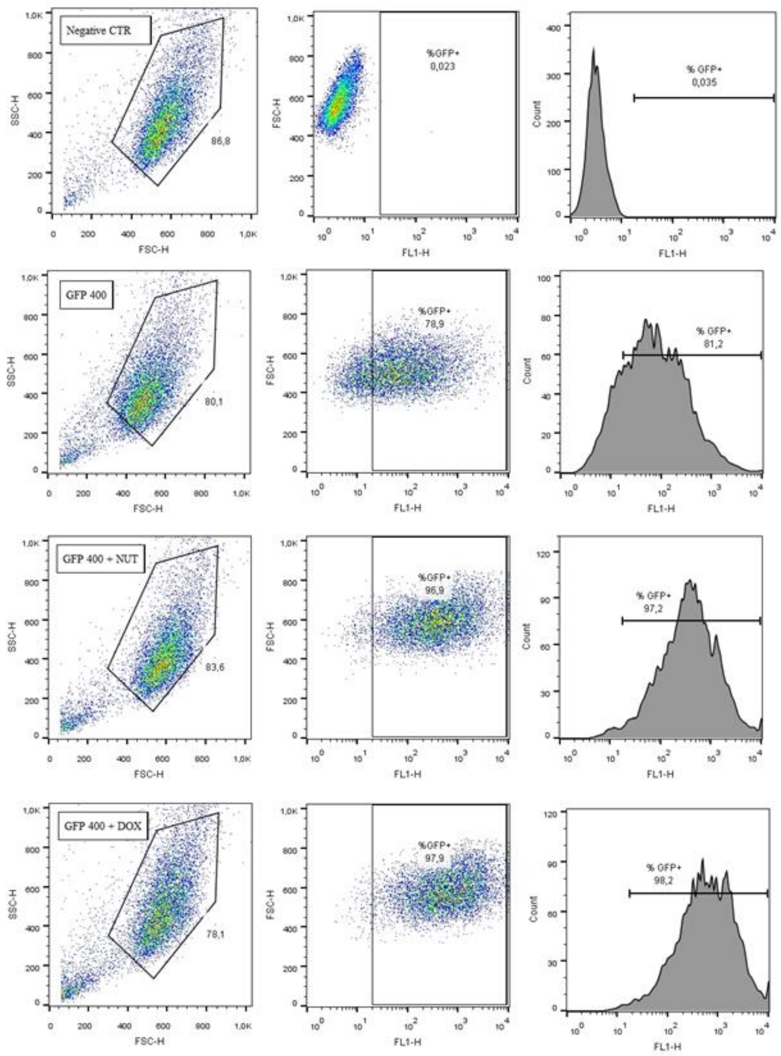

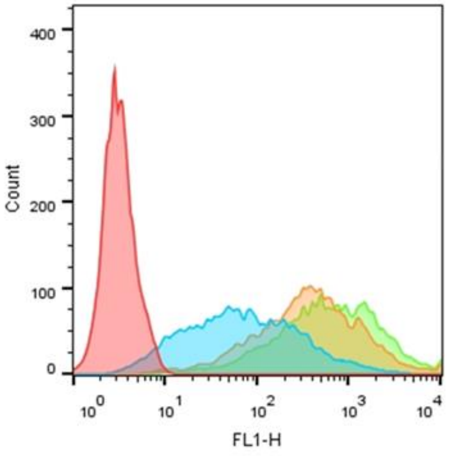

	Sample Name	Count	Geometric Mean : FL1-H
	mca ctrl neg.001	8681	3,18
	mca moi 400.004	8008	70,0
	mca moi 400 e nut.016	8355	344
	mca moi 400 e dox.010	7812	550

Figure S1

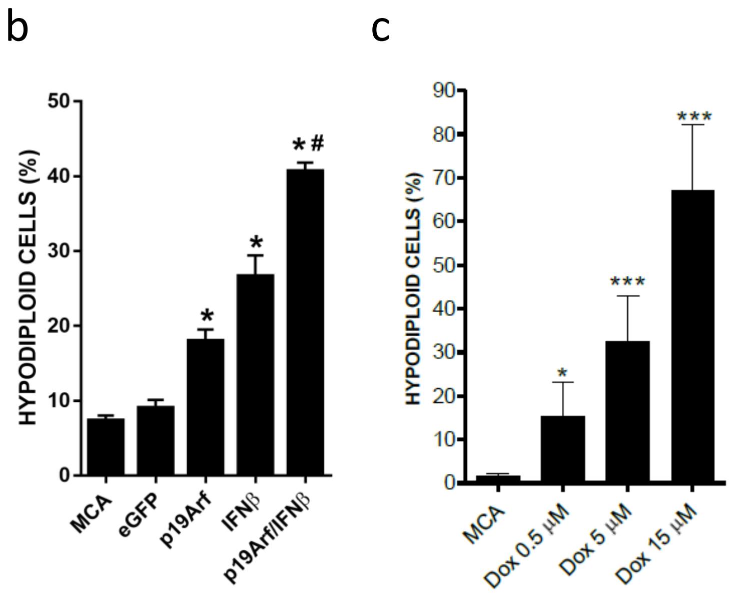

Figure S1: Characterization of MCA cells treated *in vitro*. (**a**) Cells were transduced at the indicated MOI using the AdRGD-PG-eGFP vector and then treated with doxorubicin (1mg/mL) or Nultin-3 (10µM) for 12 hours before flow cytometry analysis of the percentage of GFP positive cells and the intensity of GFP activity. Upper panel, data presented as histogram. Lower panel, representative flow cytometry data. Images generated using FlowJo software, version 10 (FlowoJo, Ashland, OR, USA, https://www.flowjo.com/). n=2. (**b**) Induction of cell death 72 hours after transduction of MCA cells with AdRGD-PG-eGFP, AdRGD-PG-p19, AdRGD-PG-IFNβ or co-transduction with AdRGD-PG-p19 and AdRGD-PG-IFNβ vectors. n=3. (**c**) Induction of cell death 72 hours after treatment of MCA cells with doxorubicin. n=3. For **b** and **c**, One-way Anova and Tukey's multiple comparision post-test. *, p<0.05, ***, p<0.001; #, p<0.05 as compared to p19ARF or IFNβ groups.

Figure S1 (continued)

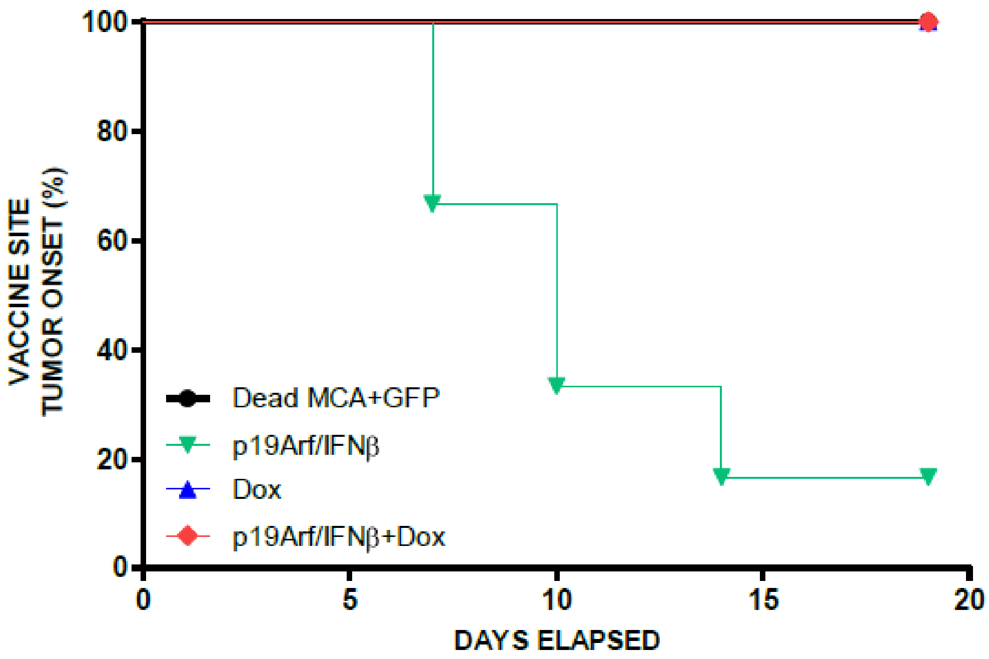

Figure S2. p19Arf/IFNβ and doxorubicin association potentiates immunogenicity of treated cancer cells. Percentage of tumor-free mice at the vaccine site after inoculation of MCA cells transduced *ex vivo* with AdRGD-PG-p19Arf and AdRGD-PG-IFNβ vectors and/or treated with Dox as per Fig. 3b of the main text.

Figure S2

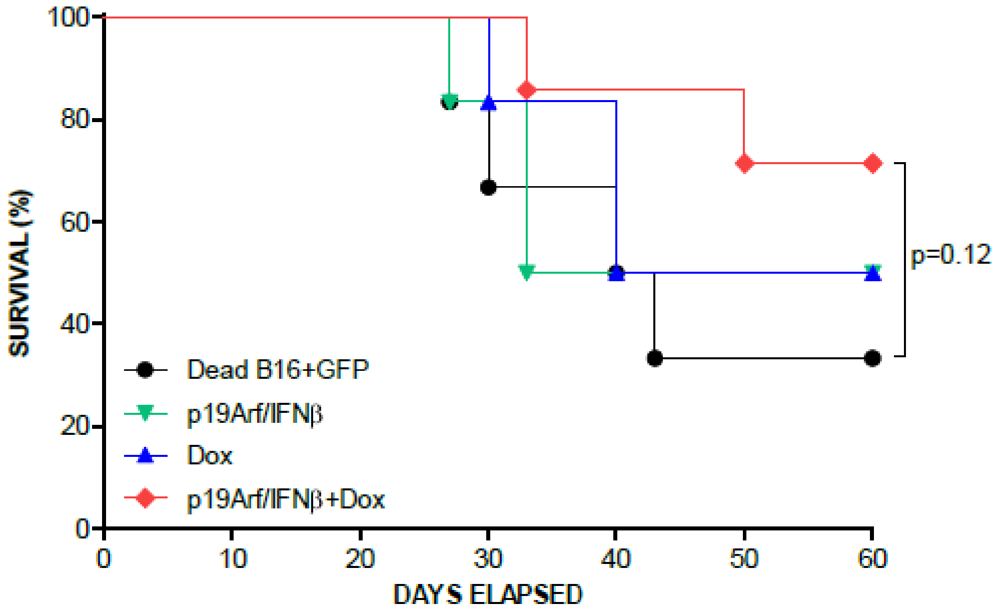

Figure S3: Association of p19Arf/IFNβ and doxorubicin potentiates immunogenicity of treated B16 cells. B16 tumors (s.c.) were established then immunotherapy performed using B16 cells treated *ex vivo* as indicated. Progression of the s.c. (challenge) tumor was monitored. Tumor survival (defined as tumor volume <1500 mm^3 and/or free of morbidity) are shown graphically. N=6 for all groups, except for p19Arf/IFNβ + Dox where n=7.

Figure S3

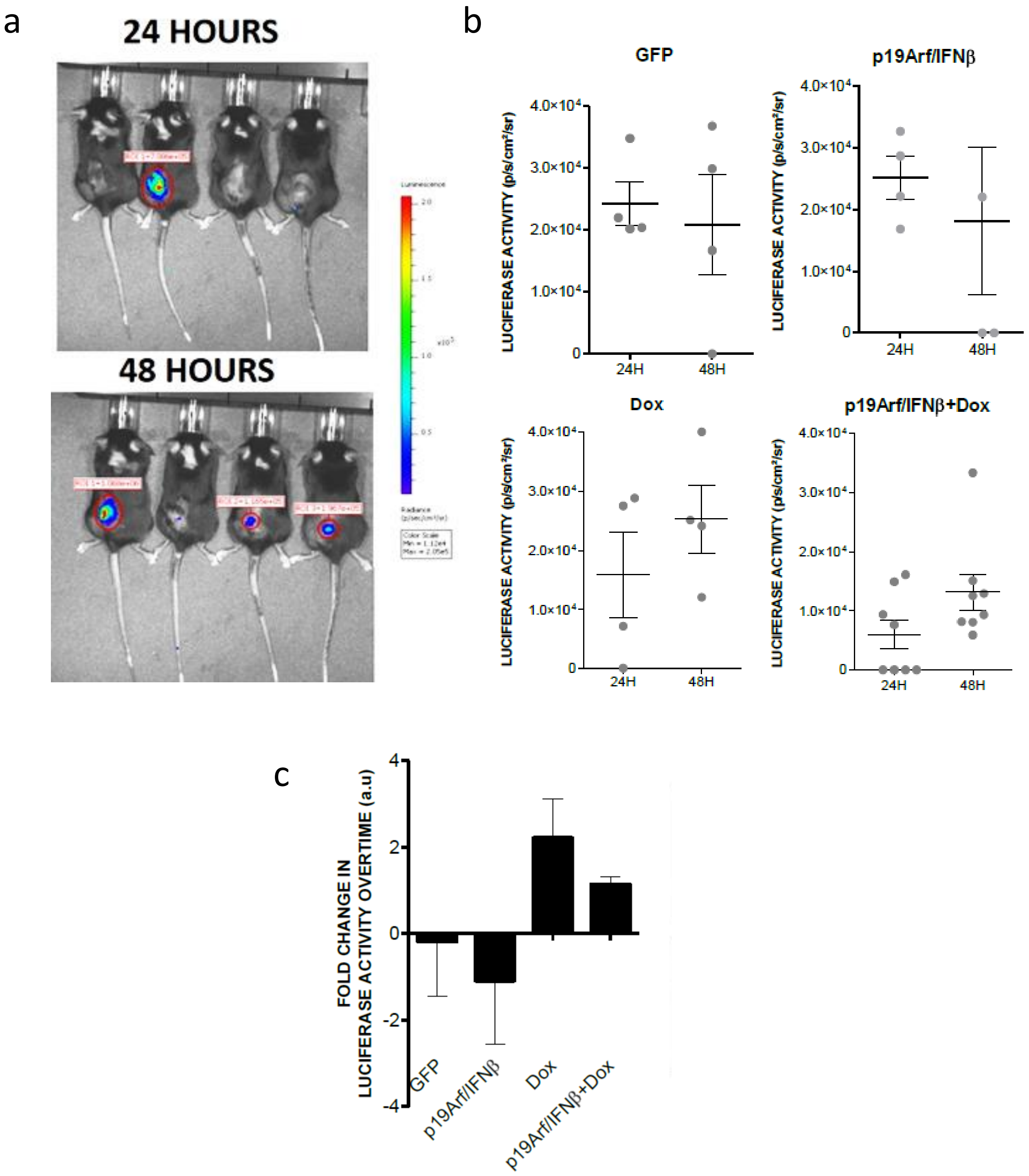

Figure S4: ***In vivo* association of p19Arf/IFNβ gene therapy with doxorubicin inhibits changes the dynamic of caspase 3 activity**. MCA-DEVD tumors (s.c.) were treated as per Figure 5 of the main text. The DEVD reporter provides luciferase activity only when caspase 3 is active, thus the assays reveals bioluminescence imaging of caspase 3-dependent luciferase activity *in vivo*. (**a**) Representative bioluminescence image of the p19Arf/IFNβ+Dox group. (**b**) Plots showing luciferase activity at the indicated time points. (**c**) Fold difference in luciferase activity comparing the 24 and 48 hours post-treatment time points measured by bioluminescence imaging of a caspase 3 reporter construct in vivo from MCA-DEVD tumors treated as mentioned in a. n = 4 for the GFP, p19Arf/IFNβ and Dox groups. n=8 for the p19Arf/IFNβ+Dox group.

Figure S4
