# Supplementary material for: Potentiation of combined p19Arf and interferon-beta cancer gene therapy through its association with doxorubicin chemotherapy
Source: Sci Rep. 2022 Aug 10;12:13636. doi: 10.1038/s41598-022-17775-y (PMC9365852; doi:10.1038/s41598-022-17775-y)
Supplement: Supplementary file 2 — Supplementary Information 2. [file 41598_2022_17775_MOESM2_ESM.pdf]

Cleaved PARP (116 kDa, 89 kDa)
MCA: Figure 2 of main text
B16: Figure 3 of main text

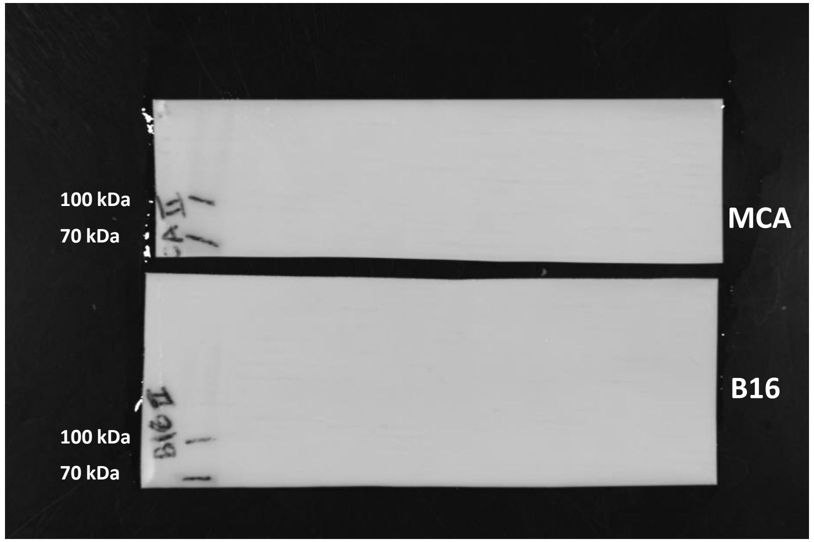

Photos of membranes

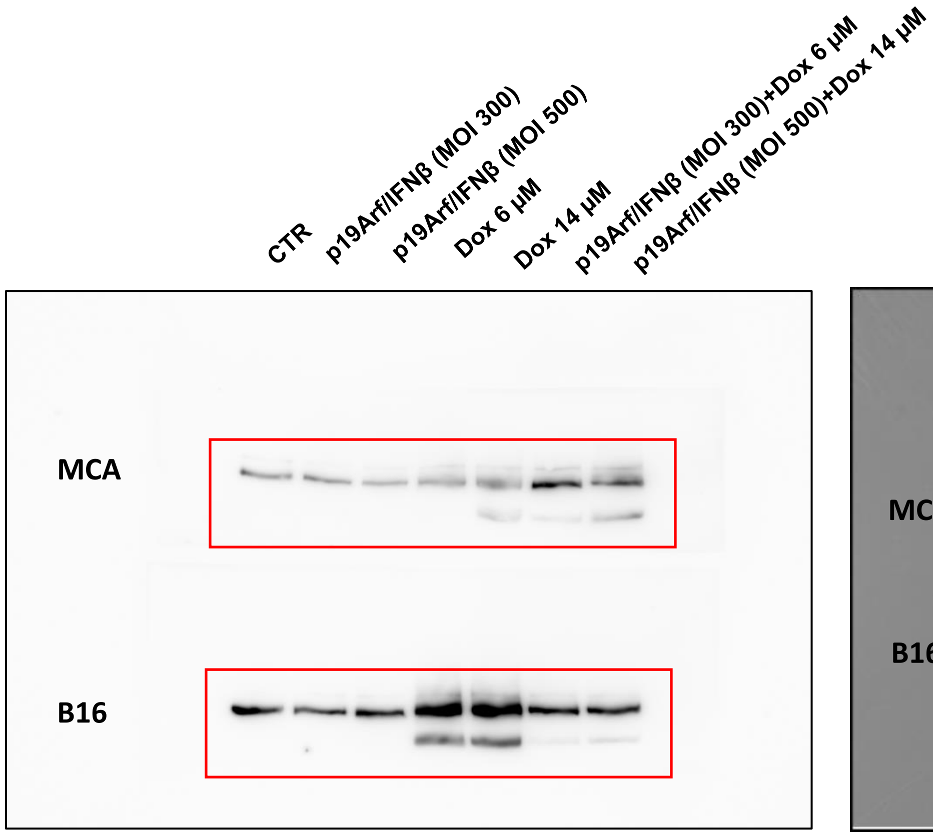

Detection
Red box: approximate area used in final figure

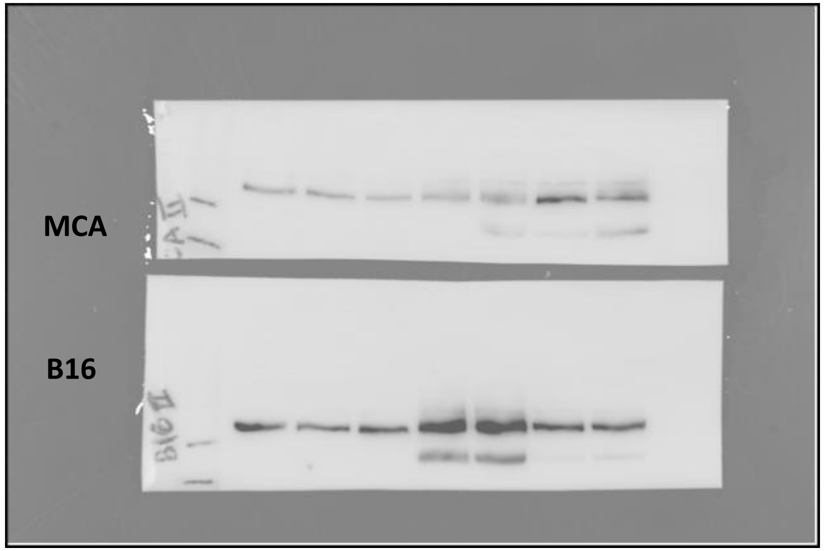

Merge

Actin (from PARP assay, 42 kda)
MCA: Figure 2 of main text
B16: Figure 3 of main text

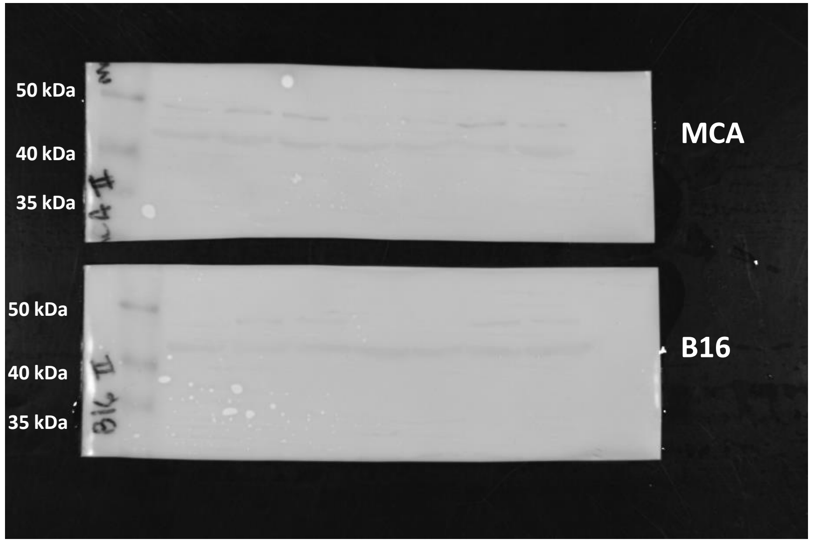

Photos of membranes

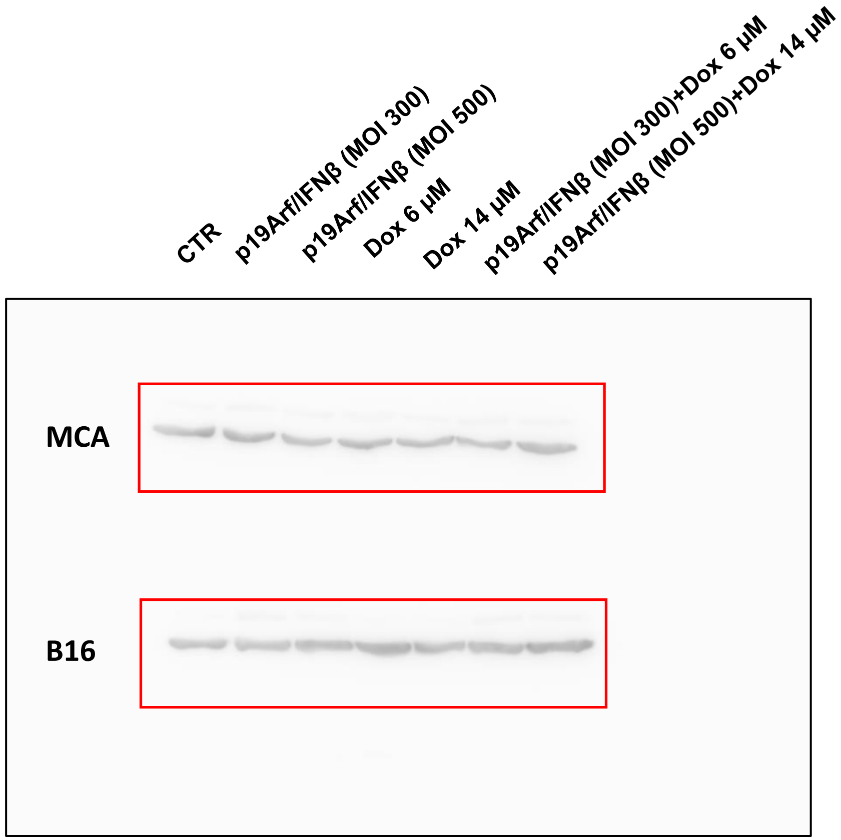

Detection
Red box: approximate area used in final figure

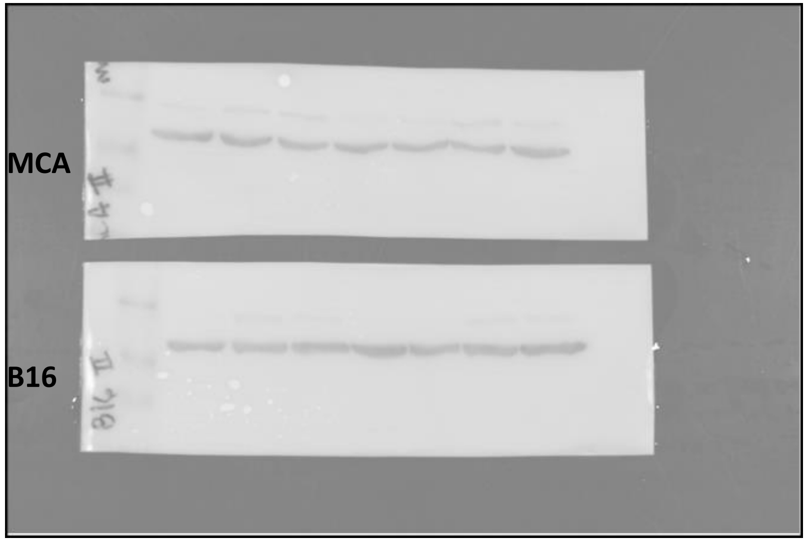

Merge

MCA
Figure 2 of main text

Caspase3 (35 kDa)

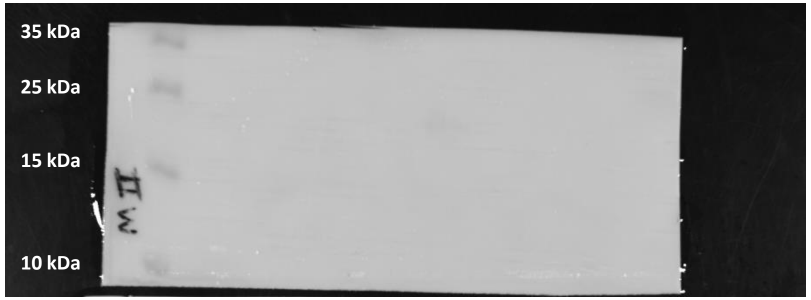

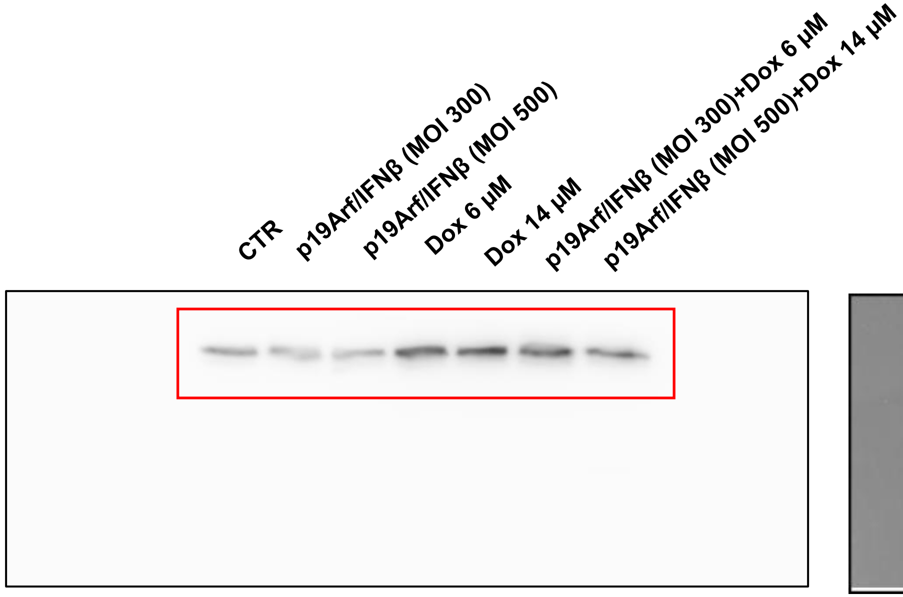

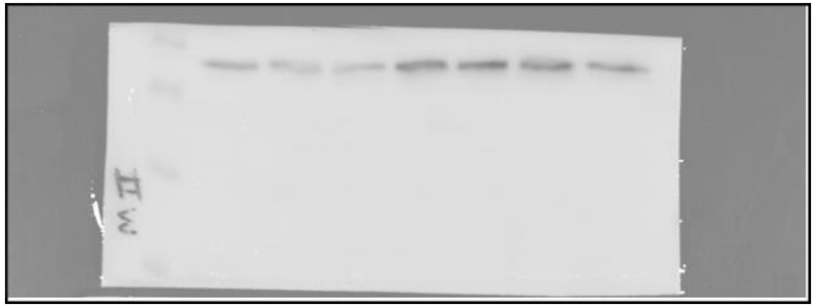

Cleaved Caspase3 (19 kDa; 17 kDa) (membrane from above was cut off at 25 kDa and re-exposed)

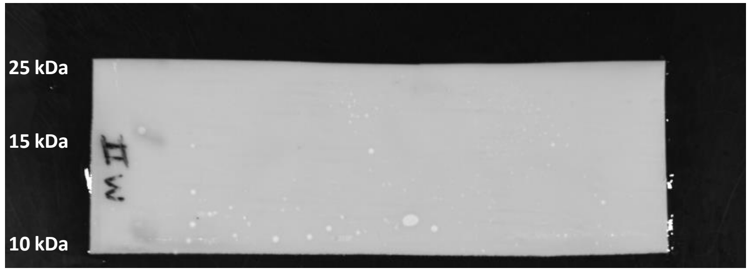

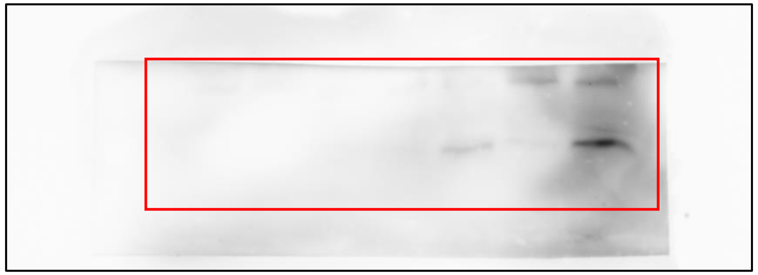

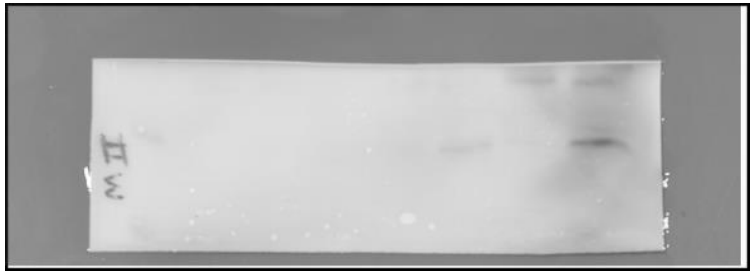

Photos of membranes

Detection
Red box: approximate area used in final figure

Merge

MCA
Figure 2 of main text

Tubulin (55 kDa) (from Caspase 3 assay)

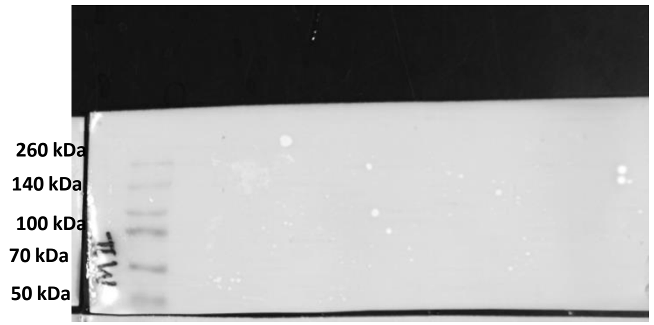

Photos of membranes

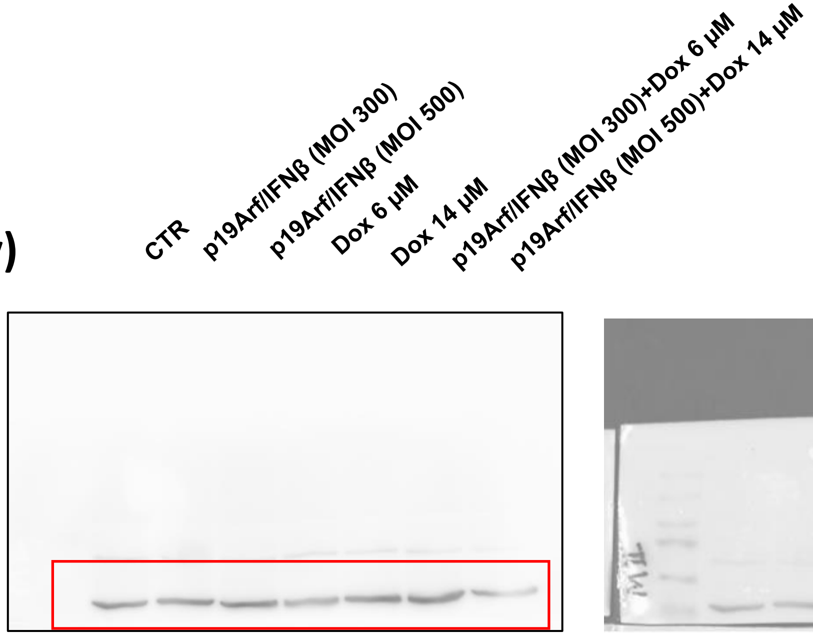

Detection

Red box: approximate area used in final figure

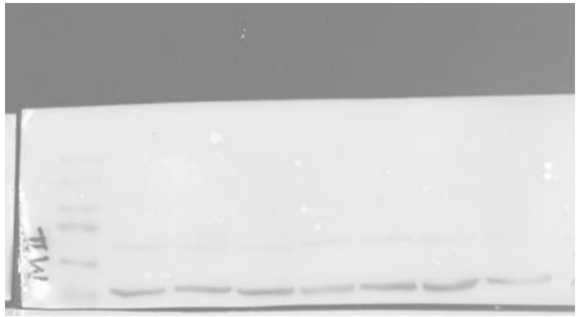

Merge

MCA
Fig 4 of main text

HMGB1 (29 kDa)

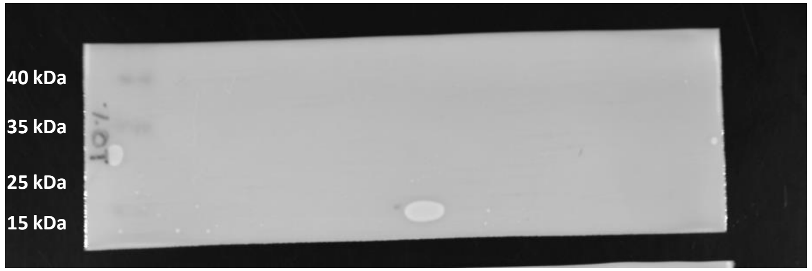

Photos of membranes

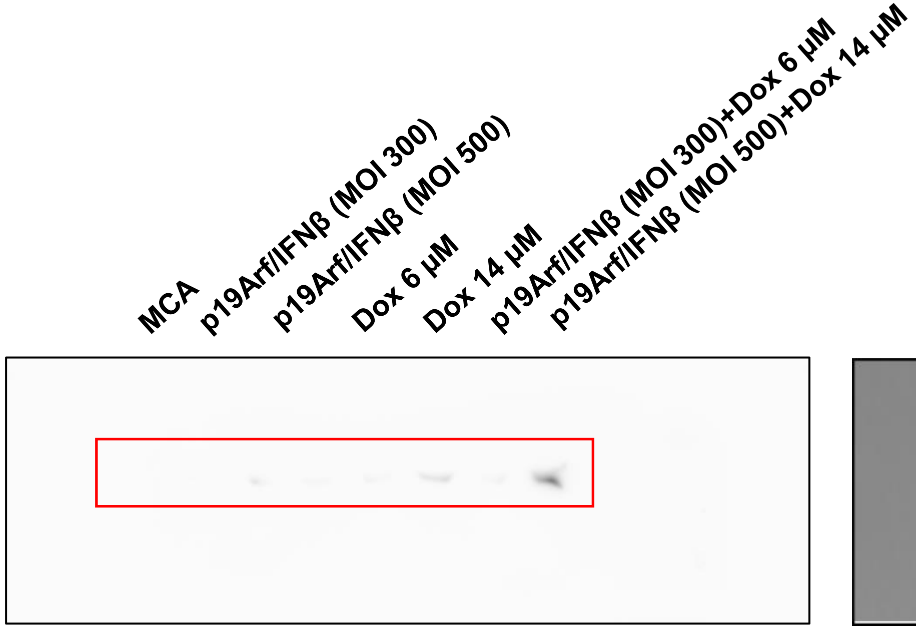

Detection
Red box: approximate area used in final figure

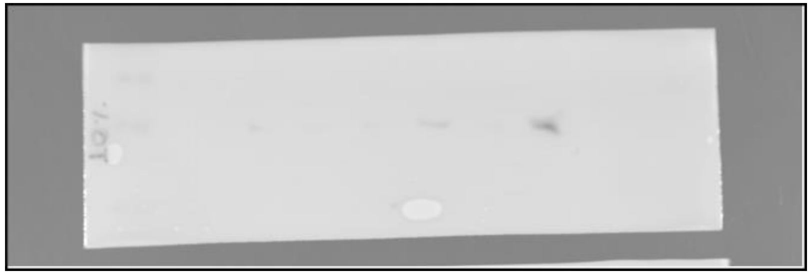

Merge

Cleaved PARP (116 kDa, 89 kDa)
Second assay (not shown in main text)

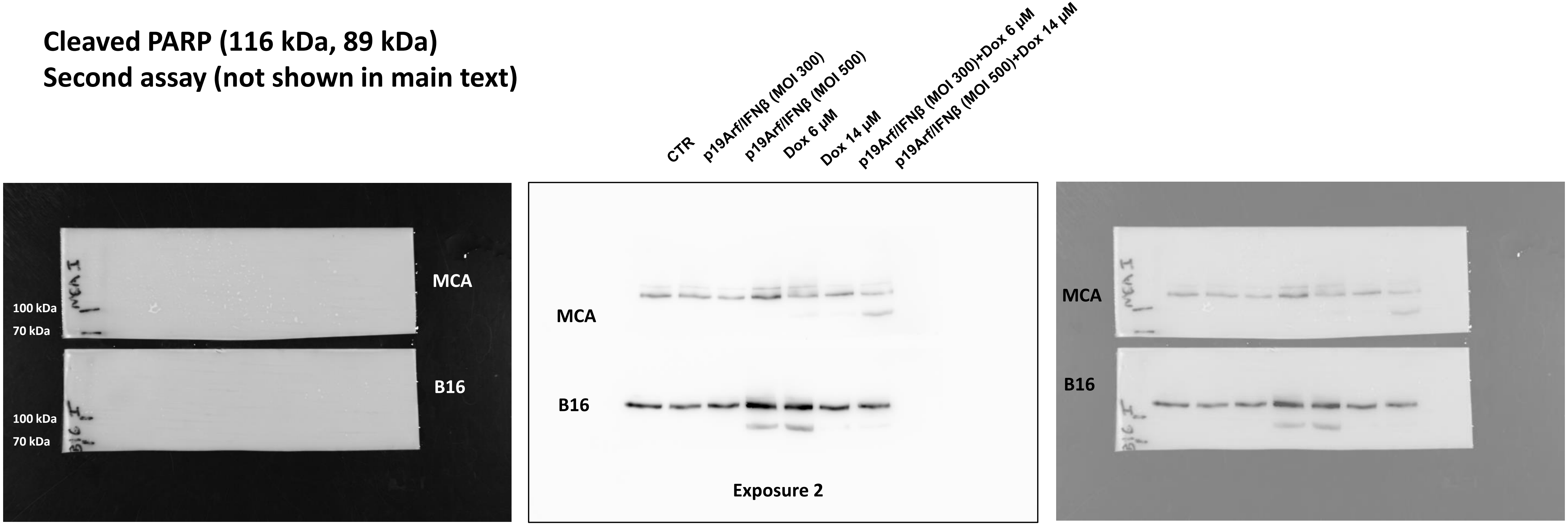

Photos of membranes | Detection | Merge

Actin (second PARP assay, 42 kDa)
Not shown in main text

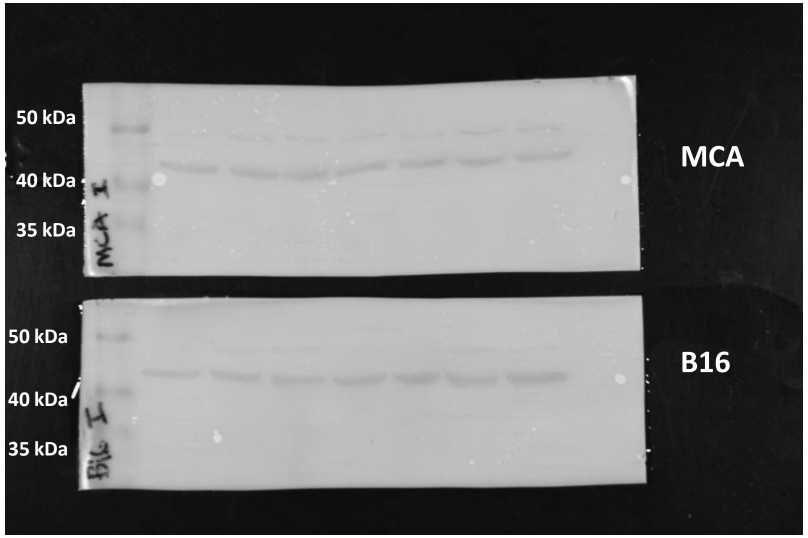

Photos of membranes

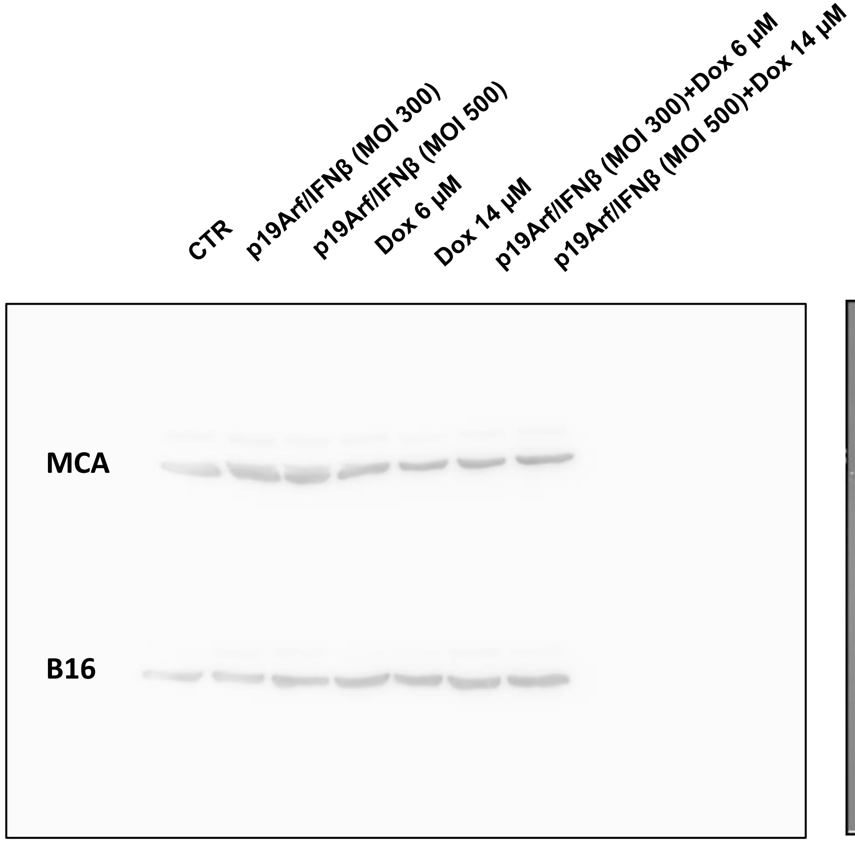

Detection

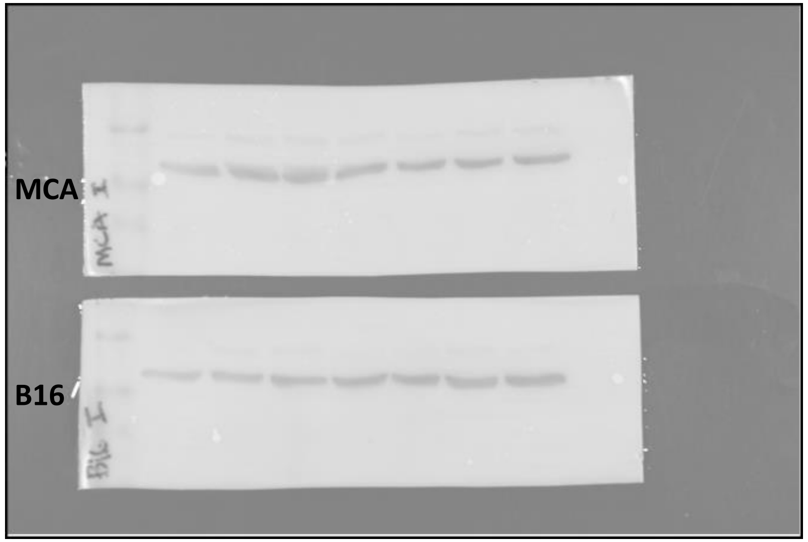

Merge

MCA
Caspase 3
Second assay, not shown in main text

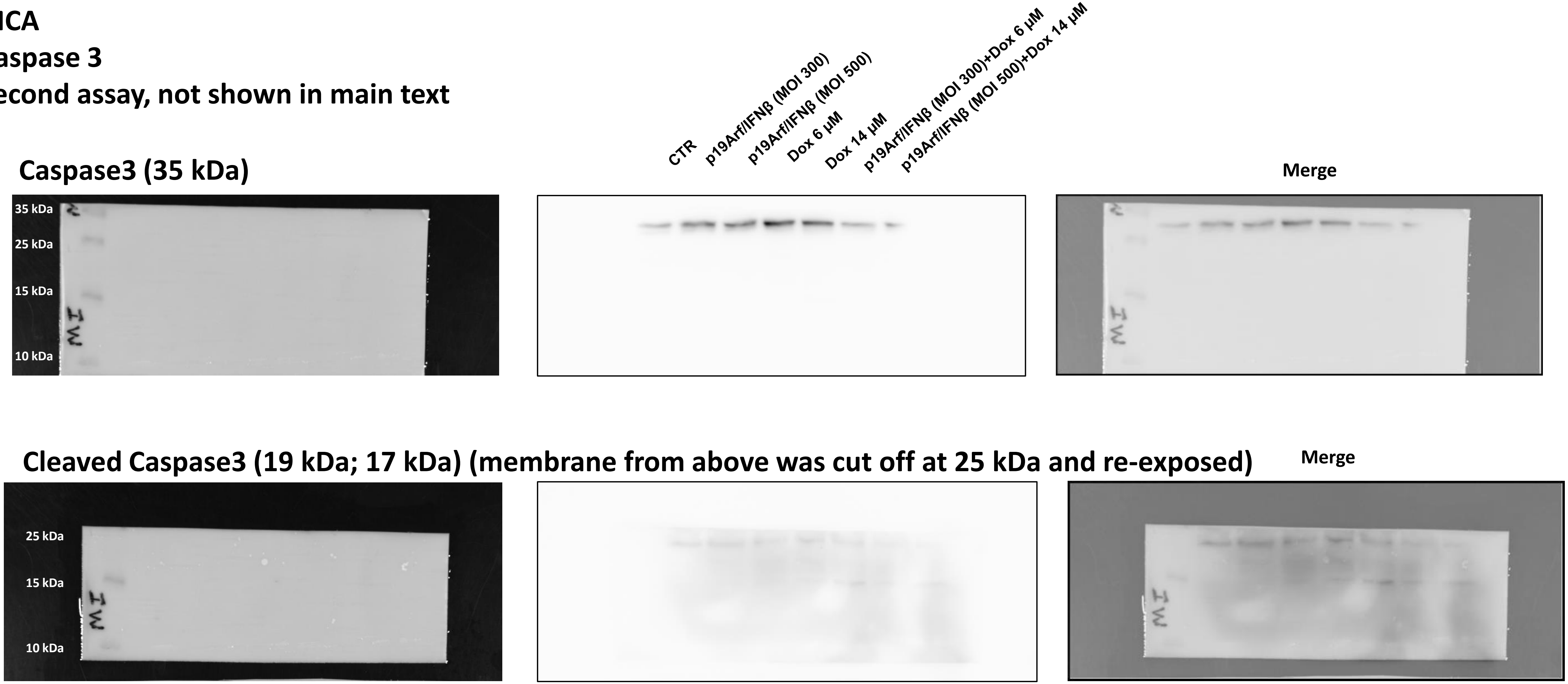

Photos of membranes | Detection | Merge

MCA
Tubulin (from second Caspase 3 assay)
Not shown in main text

Tubulin (55 kDa) (from Caspase 3 assay)

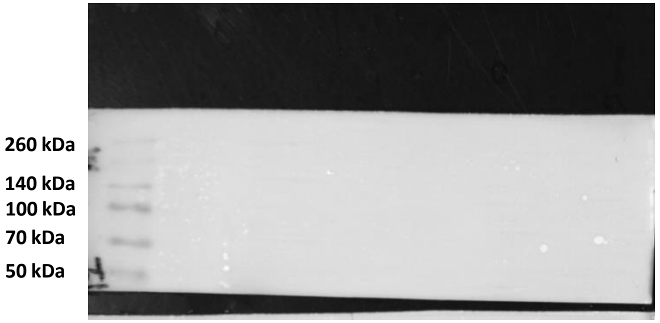

Photo of membrane

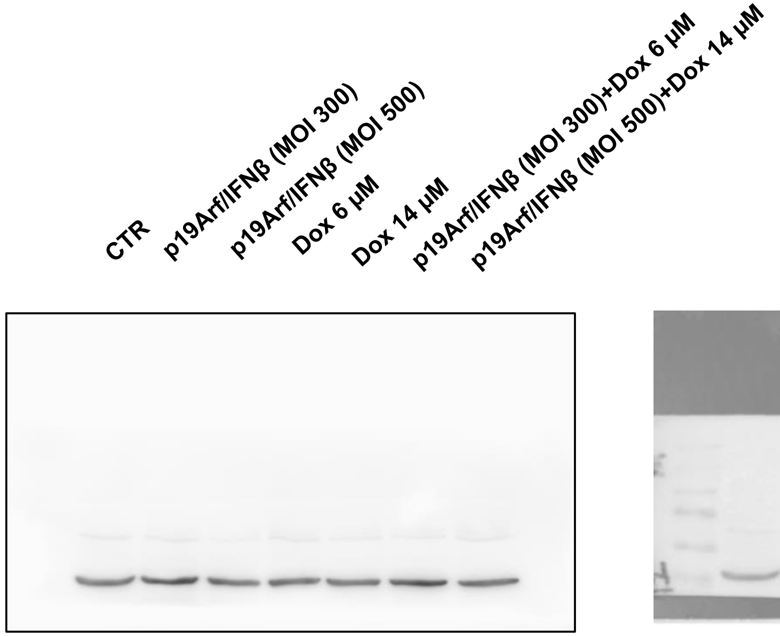

Detection

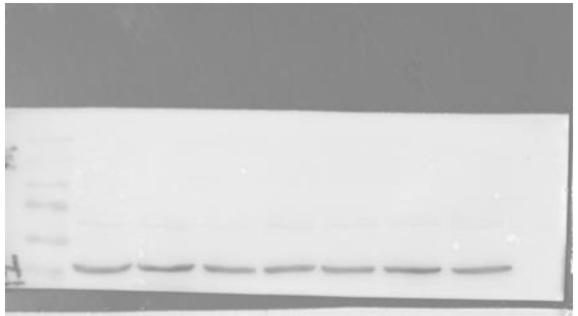

Merge

MCA
HMGB1
Second assay, not shown in main text

HMGB1 (29 kDa)

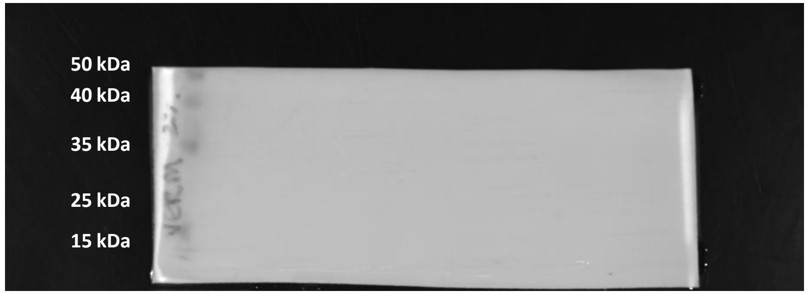

Photo of membrane

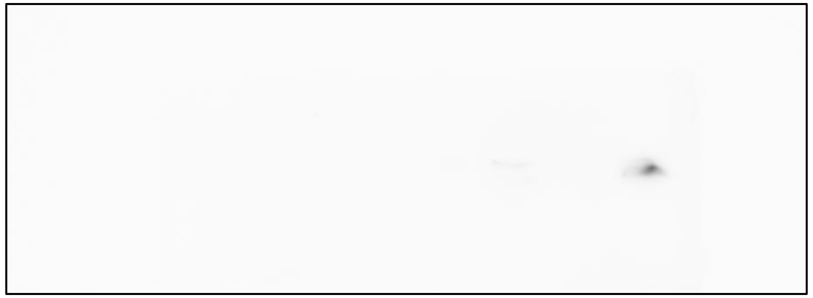

Detection

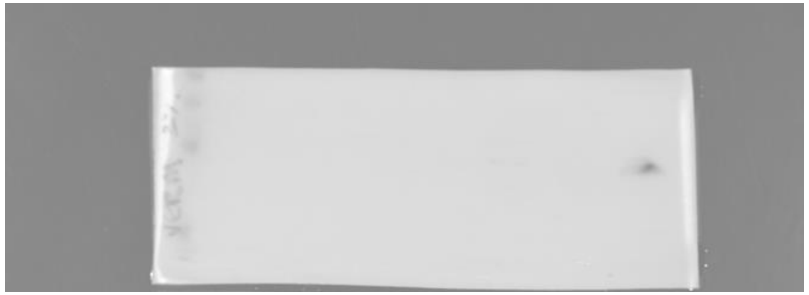

Merge

MCA
HMGB1
Third assay, not shown in main text

HMGB1 (29 kDa)

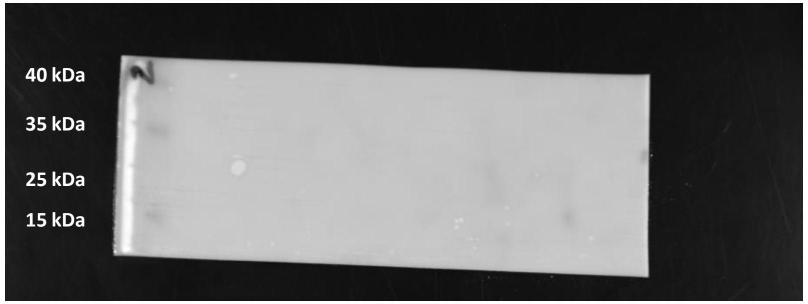

Photo of membrane

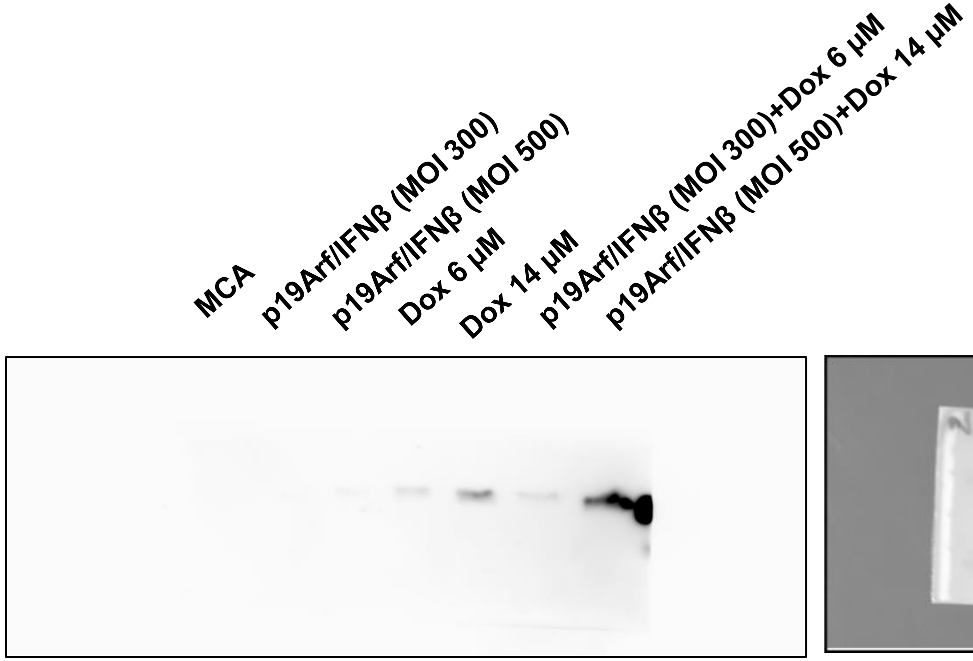

Detection

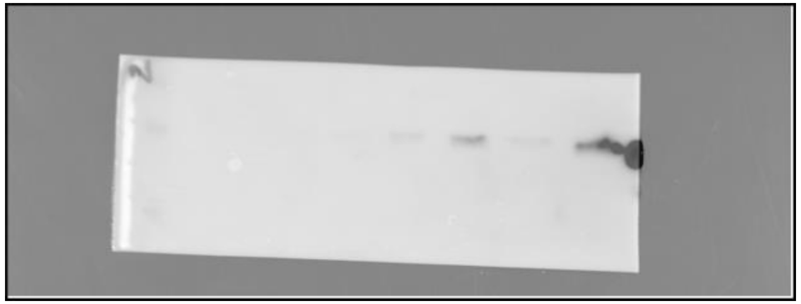

Merge
